# Inability to phosphorylate Y88 of p27^Kip1^ enforces reduced p27 protein levels and accelerates leukemia progression

**DOI:** 10.1038/s41375-022-01598-x

**Published:** 2022-05-21

**Authors:** Heidelinde Jäkel, Martin Taschler, Karin Jung, Christina Weinl, Fragka Pegka, Michael Keith Kullmann, Silvio Roland Podmirseg, Sayantanee Dutta, Markus Moser, Ludger Hengst

**Affiliations:** 1 Institute of Medical Biochemistry, Biocenter, Medical University of Innsbruck, Innsbruck, Austria; 2 Division of Hematology, Medical University of Graz, Graz, Austria; 3 Department for Molecular Medicine, Max-Planck-Institute of Biochemistry, Martinsried, Germany; 4 Institute of Experimental Hematology, School of Medicine, Technische Universität München, Munich, Germany

**Keywords:** Acute lymphocytic leukaemia, Cell signalling, Oncogenes, Oncogenesis

## Abstract

The cyclin-dependent kinase (CDK) inhibitor p27^Kip1^ regulates cell proliferation. Phosphorylation of tyrosine residue 88 (Y88) converts the inhibitor into an assembly factor and activator of CDKs, since Y88-phosphorylation restores activity to cyclin E,A/CDK2 and enables assembly of active cyclin D/CDK4,6. To investigate the physiological significance of p27 tyrosine phosphorylation, we have generated a knock-in mouse model where Y88 was replaced by phenylalanine (p27-Y88F). Young p27-Y88F mice developed a moderately reduced body weight, indicative for robust CDK inhibition by p27-Y88F. When transformed with v-ABL or BCR::ABL1^p190^, primary p27-Y88F cells are refractory to initial transformation as evidenced by a diminished outgrowth of progenitor B-cell colonies. This indicates that p27-Y88 phosphorylation contributes to v-ABL and BCR::ABL1^p190^ induced transformation. Surprisingly, p27-Y88F mice succumbed to premature v-ABL induced leukemia/lymphoma compared to p27 wild type animals. This was accompanied by a robust reduction of p27-Y88F levels in v-ABL transformed cells. Reduced p27-Y88F levels seem to be required for efficient cell proliferation and may subsequently support accelerated leukemia progression. The potent downregulation p27-Y88F levels in all leukemia-derived cells could uncover a novel mechanism in human oncogenesis, where reduced p27 levels are frequently observed.

## Introduction

p27^Kip1^ (hereafter p27) is a member of the Cip/Kip family of cyclin-dependent kinase (CDK) inhibitors. In its canonical function, it controls CDK activity and cell proliferation by binding to both subunits of a cyclin/CDK complex [1, 2]. Additional CDK-dependent and independent functions of p27 include the regulation of transcription, cell motility, cell migration, differentiation, apoptosis and autophagy [1, 3–6]. CDK inhibition by p27 depends on its posttranslational modifications and subcellular localization. p27 levels are high in quiescent cells and during early G1 phase, and decline when cells progress to S phase. They are tightly regulated by transcription, translation and endolysosomal or ubiquitin-dependent proteasomal degradation [1, 3–6].

p27 is phosphorylated by tyrosine kinases including Abelson tyrosine kinase (ABL1), Janus kinase 2 (JAK2), FMS-like tyrosine kinase 3 (FLT3), Breast Tumor Kinase (BRK) and SRC family kinases [7–11]. Tyrosine 88 (Y88) is one of three tyrosine residues within p27 which is targeted by all identified kinases. The three tyrosine residues 74, 88 and 89 in p27 are positioned within the CDK inhibitory domain. Phosphorylation of one or several sites alters CDK inhibition by p27.

p27 controls the G0/G1-phase to S-phase transition. In early G1 phase, p27 is required for the assembly of cyclin D/CDK4,6 holoenzymes [12–14]. Although p27 binding induces structural changes within the ATP-binding pocket of CDK4, the trimeric complex is inactive unless p27 is tyrosine phosphorylated [13, 15]. In its tyrosine phosphorylated form, p27 acts as an allosteric activator of cyclin D/CDK4 [12, 13, 15]. Unphosphorylated p27 can efficiently inhibit cyclin/CDK2 kinase activity. Upon Y88-phosphorylation of p27, the protein remains bound to cyclin/CDK2 complexes, but an inhibitory 3_10_-helix is ejected from the ATP-binding pocket of the catalytic cleft of CDK2 [8]. This conformational change leads to activation of the cyclin/CDK2 complex, even in the absence of CAK-mediated T160 phosphorylation of CDK2 [8, 16, 17]. The bound cyclin/CDK2 complex intramolecularly phosphorylates p27 on T187, a modification of p27 that triggers the SCF^SKP2^ E3 ubiquitin ligase-dependent proteasomal degradation of p27 [8, 17]. Activated CDK2 can initiate a feedback loop of efficient p27 degradation, that ensures low p27 levels after restriction point passage [18]. Elevated tyrosine phosphorylation of p27 might thereby contribute to hyperproliferation and oncogenesis. Low p27 levels were observed in different types of human cancer and correlate with tumor aggressiveness [1, 3–5]. Studies with p27 knock-out mice revealed that p27 is haploinsufficient for tumor suppression [19].

The oncogenic tyrosine kinases BCR::ABL1, JAK2-V617F and FLT3-ITD can cause hematological malignancies [20]. All of them phosphorylate p27 on Y88 [7–10]. The BCR::ABL1^p190^ fusion protein gives rise to B-cell acute lymphoblastic leukemia [21], which resembles a disease induced by v-ABL expression in mice. ABL oncogenes activate the PI3K-, RAS-, MYC and JAK-STAT signaling pathways and decrease p27 levels by forkhead box O (FOXO) dependent transcriptional regulation and enhanced SCF^SKP2^ dependent proteasomal degradation [21, 22].

Although p27 tyrosine phosphorylation and its molecular and structural consequences have been intensively studied, the physiological relevance in vivo remains unexplored. We generated a mouse model where tyrosine-88 of p27 was mutated to phenylalanine (Y88F), resulting in a p27 protein lacking only the hydroxyl group of Y88. Young p27-Y88F animals were smaller than wild type (WT) littermates. Compared to WT, p27-Y88F protein levels were reduced in multiple tissues from knock-in mice. In v-ABL/BCR::ABL1 transformed cells, initial transformation was impaired in p27-Y88F cells, as revealed by colony outgrowth. This is consistent with the hypothesis that Y88F might protect from transformation induced by oncogenic tyrosine kinases. Surprisingly however, v-ABL induced leukemia/lymphoma progression was strongly accelerated. Immortalized leukemia cells from p27-Y88F mice revealed strongly reduced p27 levels compared to WT cells, suggesting that the robust CDK inhibition by p27-Y88F must be compensated by decreased p27 expression.

## Material and methods

### Generation of the knock-in mice and in vivo experiments

Experiments were approved by the Austrian Federal Ministry of Science and Research (license GZ BMWFW-66.011/0033-WF/V/3b/2016). p27-Y88F knock-in mice were generated according to Besson et al. [23] and Malek et al. [24]. For in vivo leukemia studies, 100 µl of Abelson murine leukemia virus (A-MuLV) was subcutaneously injected in the neck of 1 day old mice. All new born litters from the two homozygous pairings per genotype were subjected to A-MuLV injection and subsequent analyses. Therefore, no randomization was performed. The investigator was blinded to the group when monitoring signs of disease. Sample size was estimated based on previous reports [23, 25]. A detailed description of the procedures is available in the Supplementary Methods.

### Plasmids, retroviral infection and colony formation assay

BCR::ABL1^p190^ pMSCV-IRES-GFP and v-ABL pMSCV-IRES-GFP plasmids as well as ecotropic replication-deficient A-MuLV produced by A010 cells used for injection of new born mice were a generous gift of Veronika Sexl (Veterinary University of Vienna, Austria). Colony formation was assessed in duplicates in semisolid methylcellulose (MethoCult M3234, Stem Cell Technologies, Vancouver, USA). A detailed method description is provided in the Supplementary Methods.

### Generation of stable HeLa Flp-In T-REx cell lines

Tyrosine 88 of p27 was mutated to phenylalanine in pDONR207 WT p27 or in the cyclin/CDK-binding deficient variant p27-CK^-^ [26] by Quickchange mutagenesis (Agilent, St. Clara, CA, USA) and recombined with the pTO-GW-FRT destination vector (generated by Stephan Geley (Innsbruck, Austria)). Stable integration into HeLa Flp-In T-REx cells (obtained from Stephan Geley) was achieved by cotransfecting destination vectors with pOG44 (Invitrogen, fisher scientific, Waltham, M, USA) followed by 200 μg/ml hygromycin B (Merck, Darmstadt, Germany) selection for 10 days. All constructs have been verified by sequencing.

### Immunoblotting and flow cytometry

Immunoblot analysis was performed as described [9]. Tissues were ground into powder in liquid nitrogen and suspended in tissue-extraction buffer (1% Triton X-100, 150 mM NaCl, 50 mM HEPES pH 7.5, 1 mM EDTA, 1 mM EGTA,10% glycerol, 0.25% sodium deoxycholate, 1% SDS, Phosstop (Roche, Mannheim, Germany) and protease inhibitor cocktail (Sigma-Aldrich, St. Louis, M, USA), sonicated (Bandelin Sonopuls ultrasonic homogenizer, Sigma-Aldrich) and centrifuged at 24,000 × *g* for 20 min. Cells from cell culture were washed with ice cold PBS and suspended in 2x Lämmli buffer (100 mM Tris [pH 7.4], 20% glycerol, 4% SDS) and sonicated. Protein concentration was determined using the DC Protein Assay (Bio-Rad, Hercules, CA, USA). The signals were detected using the Clarity detection reagent (Biorad), the ECL Prime or Select detection reagents (Cytiva, Emeryville, CA, USA) and the LAS4000 imaging apparatus (Cytiva). The mouse anti-p27^Kip1^-HRP conjugated antibody (clone 57, Becton Dickinson Biosciences, Franklin Lakes, NJ, USA) was used for the detection of p27, except for HeLa Flp-In cell extracts where the rabbit anti-p27^Kip1^ antibody (C19, sc-528, St. Cruz Biotechnology, Texas, USA) was employed. A detailed list of antibodies and the description of flow cytometry analyses are provided in the Supplementary Material and Methods.

### Quantitative RT-PCR (qPCR)

Total RNA from cells or snap frozen grounded mouse tissues was extracted using the RNeasy Mini Kit (Qiagen, Hilden, Germany). Reverse transcription was performed with the RevertAid Reverse transcriptase (Fisher scientific) following the manufacturer’s instructions. All qPCRs were performed in triplicates with the SensiFAST SYBR No-ROX Kit (Bioline, Meridian Bioscience, London, UK) on a Rotor Gene Q cycler (Qiagen). mRNA expression was normalized and expressed relative to the endogenous reference gene TATA box binding protein (TBP): fold induction = 2(−ΔCt), where ΔCt = Ct(p27)–Ct(TBP). Primer sequences and cycling conditions are listed in the Supplementary Material and Methods.

### Statistical analysis

Two-tailed unpaired Student’s *t* test was carried out when comparing two groups unless otherwise specified. Normal distribution was tested by the Kolmogorov–Smirnov Test and similarity of variances by the *F*-test. Survival was represented with a Kaplan–Meier curve and statistical significance was determined with the log-rank test. Data were analyzed by GraphPad Prism 8 software (GraphPad Software, San Diego, CA, USA) or SPSS software (IBM, Chicago, IL, USA) and are represented as mean ± standard deviation unless otherwise specified. Statistical significance (*p < 0.05, **p < 0.01, ***p < 0.001, ****p < 0.0001) and sample size are indicated in each figure legend.

## Results

### Body weight is moderately reduced in young p27-Y88F mice

**Fig. 1:**
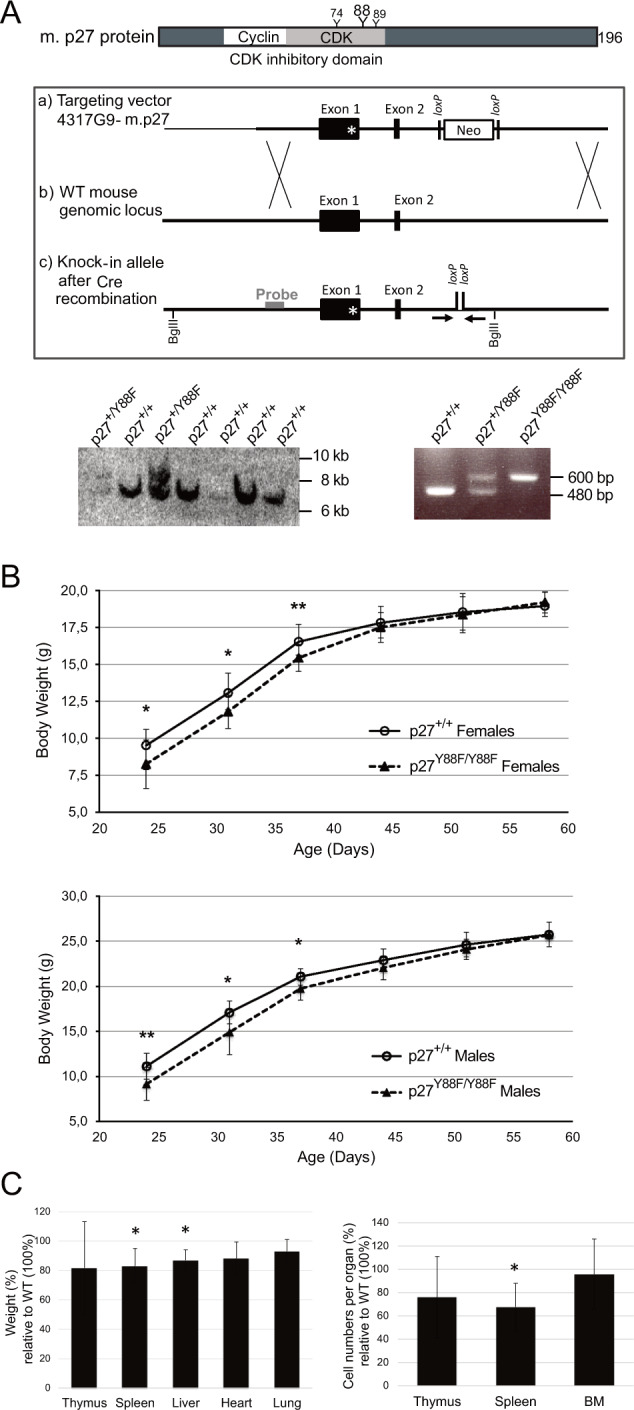
Young p27-Y88F KI mice are characterized by reduced size. **A** Generation of p27-Y88F KI mice. Upper panel: schematic representation of mouse (m.) p27 and the location of the three tyrosines in the CDK-binding domain. Middle panel: targeting strategy to replace wild type p27 with the p27-Y88F mutated allele. Homologous recombination of the targeting vector containing the Y88F mutation of p27 in Exon 1 as indicated by a star (a) and the wild type (WT) p27 mouse locus (b) is depicted. (c) Targeted knock-in allele after excision of the Neomycin cassette by Cre recombinase. Primers used for genotyping are indicated as arrows and the probe and the BglII sites used for Southern blot screening are shown. Southern blot screening of electroporated ES cells (lower left panel). The 7.3 kb fragment corresponds to the p27 wild type allele and the 8.9 kb fragment to the p27-Y88F mutant allele comprising the Neomycin cassette. Lower right panel displays genotyping results of p27 wild type (480 bp) and targeted p27-Y88F knock-in (600 bp) mice using the primers indicated above. **B** Body weight of female (upper panel) and male (lower panel) p27^+/+^ and p27-^Y88F/Y88F^ mice plotted as a function of age. Mean ± SD of 11 wild type and 16 p27-Y88F females and 9 wild type and 15 p27-Y88F males is indicated (*p < 0.05, **p < 0.01). **C** Organ weight (left panel) and cell counts from hematopoietic organs (right panel) of 40 days old p27-Y88F knock-in females relative to wild type littermates are represented as mean ± SD (*n* = 5 per group, each group includes littermates from two independent expriments). An independent experiment was performed in males. One sample *t*-test was performed by SPSS statistics (*p < 0.05).

Phosphorylation of tyrosine residue 88 (Y88, Fig. 1A upper panel), can convert the CDK inhibitor into an activator of CDK [8, 13, 15]. To investigate the physiological significance of p27-Y88 phosphorylation, we generated knock-in mice in which Y88 was exchanged to the non-phosphorylatable amino acid phenylalanine (p27-Y88F) (Fig. 1A middle panel). Neomycin resistant embryonic stem cells transduced by the p27-Y88F targeting vector were identified by Southern blot (Fig. 1A, lower left panel). The neomycin cassette was excised by breeding with Sox2-Cre mice [27]. WT and p27-Y88F knock-in mice were identified by genotyping (Fig. 1A, lower right panel). The progeny from heterozygous p27-Y88F pairings was recovered with approximately the expected Mendelian ratio (Supplementary Table 1), suggesting that this single point mutation does not interfere with essential developmental processes.

After weaning both, homozygous p27-Y88F females and males, a significantly lower body weight was monitored compared to their WT counterparts (mean difference at day 24: 17.7% in males and 13.3% in females), which assimilated after 6–8 weeks to the weight of their WT littermates (Fig. 1B). Organ weight was still decreased in 40 days old p27-Y88F mice, with a most pronounced average reduction of 18% (*p* = 0.029) in thymus and 17% (*p* = 0.012) in spleen (Fig. 1C left panel). Also average cell numbers were decreased, by 24% in thymus (*p* = 0.19) and by 33% in spleen (*p* = 0.023) (Fig. 1C right panel). These observations are consistent with the hypothesis that Y88 phosphorylation of p27 supports cell proliferation.

### Normal development of hematopoietic lineages in p27-Y88F mice

**Fig. 2:**
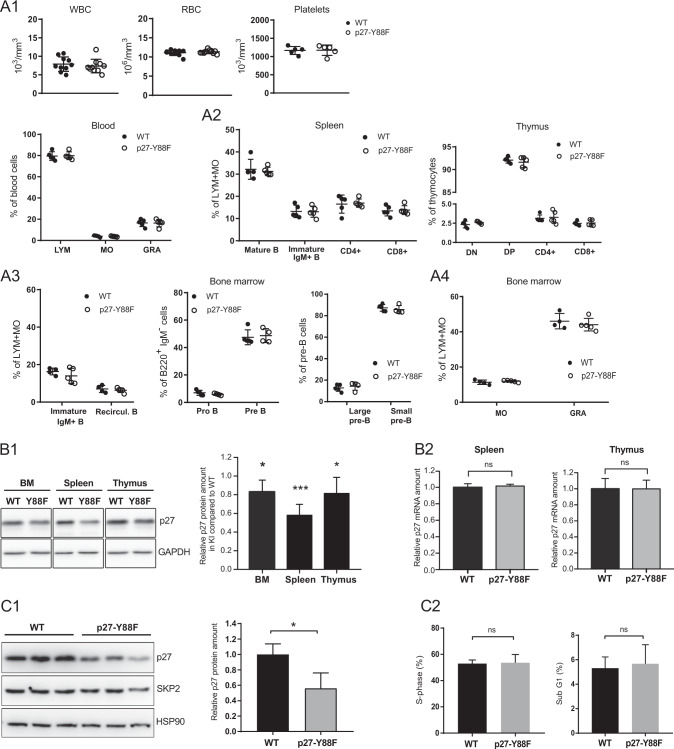
p27 levels are decreased in hematopoietic cells of p27-Y88F mice whereas hematopoietic cell populations and early B-cell development remain unchanged. **A** Characterization of hematopoietic populations. **A**1 Analysis of white blood cell count (WBC), red blood cell count (RBC), platelet counts (Platelets, upper panels) and lymphocytes (LYM), monocytes (MO) and granulocytes (GRA) (lower panel) with the ABC hemocytometer (*n* = 10 per group for WBC and RBC, *n* = 5 per group for platelets, LYM, MO and GRA). **A**2 Flow cytometry analysis of T and B-cell populations in the spleen (left panel). Frequencies of mature B cells (IgD^+^ IgM^+^), immature IgM^+^ B cells (IgM^+^ IgD^low^) CD4^+^ T cells (CD3^+^CD4^+^CD8^−^) and CD8^+^ T cells (CD3^+^CD8^+^CD4^−^) of wild type and p27-Y88F mice are represented. Flow cytometry analysis of thymocyte subsets in the thymus (right panel). Double negative thymocytes (DN) (CD4^−^CD8^−^), double positive thymocytes (DP) (CD4^+^CD8^+^), CD4^+^ T cells (CD8^−^CD4^+^) and CD8^+^ T cells (CD8^+^CD4^−^) of wild type and p27-Y88F mice are depicted. (*n* = 5 per group); **A**3 Flow cytometry analysis of bone marrow subsets in wild type and p27-Y88F mice (*n* = 5 per group). Frequencies of individual populations during early B-cell development have been analyzed as indicated. Left panel shows immature IgM^+^ B cells (B220^low^IgM^+^) and recirculating B cells (B220^high^IgM^+^). In the central panel pro-B cells (B220^+^IgM^−^CD25^−^c-kit^+^) and pre-B cells (B220^+^IgM^−^CD25^+^c-kit^−^) and in the right panel discrimination of the two pre-B subpopulations large pre-B and small pre-B cells by FS and SSC is represented. **A**4 Frequencies of monocytes (MO) (CD115^+^) and granulocytes (GRA) (CD115^−^SSC^+^) in the bone marrow are displayed. Data are represented as mean ± SD. No significant difference was detected between 10 weeks old wild type and knock in mice. **B** Analysis of p27 protein and mRNA levels in haematopoietic organs of 10 weeks old wild type and p27-Y88F mice. Littermate pairs stem from three different breedings (*n* = 5 per genotype). **B**1 Representative western blots of bone marrow (BM), spleen and thymus. Protein levels of p27 and GAPDH as loading control are represented. The right panel shows quantification of p27 protein normalized to GAPDH in p27-Y88F mice. p27 levels from wild type tissues have been set to 1. One sample *t*-test was used for statistical analysis. **B**2 qPCR analysis of p27 mRNA reveals no significant difference between splenocytes (left panel) and thymocytes (right panel) from wild type and p27-Y88F mice. **C** Immunoblot analysis of progenitor B-cell enriched cell cultures. Bone marrow cells were isolated from wild type and p27-Y88F mice and cultivated in B-cell culture medium containing 10 ng/ml rhIL7 for 7 days. *n* = 3 mice per group; **C**1 Protein levels of p27, SKP2 and HSP90 as loading control are represented. Right panel shows quantification of p27 protein normalized to HSP90. **C**2 Bar graphs represent percentage of wild type and p27-Y88F cells in S phase (left panel) and sub-G1 phase (right panel) analyzed by flow cytometry. Bar graphs represent mean ± SD. (*p < 0.05, ***p < 0.001).

To investigate if hematopoiesis is affected in p27-Y88F animals, we determined the hematopoietic populations. Macroscopic appearance and histological analyses of spleen and bone marrow did not reveal any significant differences (Supplementary Fig. 1). Similarly, the peripheral blood parameters white blood counts, red blood counts or platelets (Fig. 2A1) as well as percentages of lymphocytes, monocytes and granulocytes (Fig. 2A1 bottom panel) were comparable to WT littermates. Flow cytometric analyses indicated that p27-Y88F does not interfere with B or T cell maturation in spleen or thymus (Fig. 2A2). Detailed analysis of bone marrow cells revealed that the differentiation of B cells (Fig. 2A3) and myeloid populations (Fig. 2A4) remain largely unaffected in p27-Y88F mice. This suggests that the development of hematopoietic blood cell lineages and B-lymphopoiesis is not essentially altered in p27-Y88F mice.

### p27 levels are reduced in p27-Y88F mice

One consequence of p27-Y88 phosphorylation is its SCF^SKP2^-dependent degradation [1, 8]. Accordingly, we speculated that p27-Y88F levels might be increased. Relative p27 levels were determined in haematopoietic organs of WT and p27-Y88F mice. Surprisingly, we observed reduced p27-Y88F levels in single cell suspensions of hematopoietic organs of 10 weeks old p27-Y88F mice when compared to WT littermates (Fig. 2B1). p27-Y88F levels were also reduced in other organs (unpublished observations). To determine the levels of p27-Y88F in proliferating cells, we cultured isolated bone marrow cells for 7 days in the presence of IL-7. Again, we observed a significant downregulation of p27-Y88F protein levels to 44% in these B-cell enriched cultures (Fig. 2C1). Despite the significantly decreased p27-Y88F levels, the cell cycle phase distribution remained unaltered (Fig. 2C2 left panel and Supplementary Fig. 2). In addition, no significant change in apoptosis (sub-G1 phase cells) was observed (Fig. 2C2 right panel). These data reveal an unexpected and reproducible decrease of p27-Y88F levels in the knock-in mice, indicating a strong selective pressure to reduce p27-Y88F levels in tissues and proliferating cells.

This decline is not based on transcriptional regulation, as p27 mRNA levels from spleen and thymus did not elicit any significant difference between WT and p27-Y88F cells (Fig. 2B2). In human cancer, p27 levels are frequently reduced by SKP2 overexpression [28]. However, SKP2 protein expression in cells from bone marrow, spleen or thymus revealed no significant difference between WT and p27-Y88F knock-in cells (Supplementary Fig. 3 and Fig. 2C1). This excludes that the downregulation of p27-Y88F protein levels in knock-in mice takes place at the transcriptional level and indicates a mechanism independent of SKP2 deregulation.

### p27-Y88F mice succumb earlier to v-ABL induced B-cell leukemia/lymphoma

**Fig. 3:**
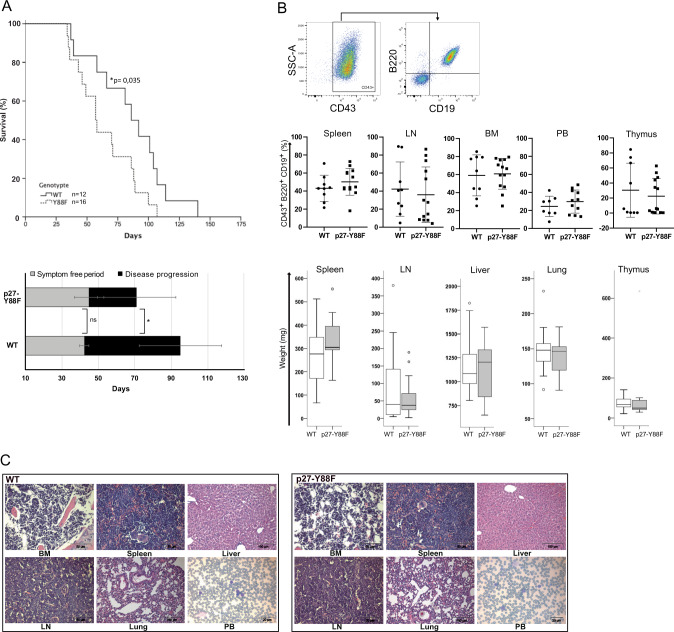
p27-Y88F mice succumb earlier v-ABL induced leukemia. **A** Kaplan–Meier curve illustrates survival of p27-Y88F and wild type mice after A-MuLV injection (*n* = 12 wild type, *n* = 16 p27-Y88F, log-rank test: *p = 0.035). Lower panel represents stages of disease of divided into symptom free period (gray bars, period of time until first signs of disease were observed) and disease progression (black bars, period of time calculated from onset of disease until occurrence of detrimental health problems followed by euthanasia) (*n* = 11 wild type, *n* = 13 p27-Y88F). **B**, **C** End point analyses of mice represented in the Kaplan–Meier curve. **B** Upper panel represents dot blots from p27-Y88F BM, illustrating the gating strategy. Viable cells are the mother population of the first gate. The black arrow indicates further gating. Middle panel: percentages of CD19^+^B220^+^CD43^+^ leukemic progenitor B cells in bone marrow (BM), LN (lymph node), PB (peripheral blood) spleen and thymus of wild type and p27-Y88F mice are represented. Graphs display mean ± SD. Lower panels display total weight of spleen, lymph nodes (LN), thymus, liver and lung from wild type and p27-Y88F leukemic mice as Box-Whisker plots. Center value represents median, the box 25th to 75th percentiles and whiskers min to max. Dots represent outliers. **C** Representative histological sections of bone marrow (BM), spleen, liver, lymph node (LN), lung and peripheral blood (PB) from leukemia bearing wild type and p27-Y88F mice. Scale bars represent 50 µm in BM, spleen and LN, 100 µm in liver and lung and 20 µm in peripheral blood.

Our starting hypothesis was, that p27-Y88F mice might be protected from tyrosine kinase-induced oncogenesis, since it would confer resistance to p27 inactivation by its tyrosine phosphorylation. To test this hypothesis, we used a mouse model that induces highly proliferative B-cell leukemia/lymphoma [29]. Expression of v-ABL in this system efficiently phosphorylated p27 on Y88 [8] (Supplementary Fig. 4). Unexpectedly, p27-Y88F mice died significantly earlier (median of 57 days, 95% CI 45–68) than WT mice (median of 86 days, 95% CI 67–104) (Fig. 3A). The symptom free period was similar in both groups (42.30 ± 2.5 days and 44.75 ± 8.1 days) (Fig. 3A, lower panel, gray bars). Subsequently, p27-Y88F mice showed severely accelerated disease progression with an average of 26.08 ± 21.7 days compared to 52.6 ± 22.6 days in the WT group (Fig. 3A, lower panel, black bars). The development of v-ABL induced B-cell leukemia/lymphoma was confirmed in all mice by the presence of characteristic B220^+^/CD19^+^/CD43^+^ leukemic progenitor B cells [29] (Fig. 3B), except for one WT mouse which developed CD4^+^CD8^-^ T cell leukemia. No significant alterations were detected between diseased WT and p27-Y88F mice regarding the infiltration rates of leukemic cells (Fig. 3B, middle panels), leukemic cell counts (Supplementary Fig. 5), the weight of the hematopoietic tissues (Fig. 3B, bottom panels) and their morphological or histological structure (Fig. 3C). Together these data demonstrate that p27-Y88F mice develop characteristic v-ABL induced progenitor B-cell leukemia/lymphoma and are prone to accelerated disease progression.

### p27-Y88F protects from initial transformation by v-ABL and BCR::ABL1^p190^

**Fig. 4:**
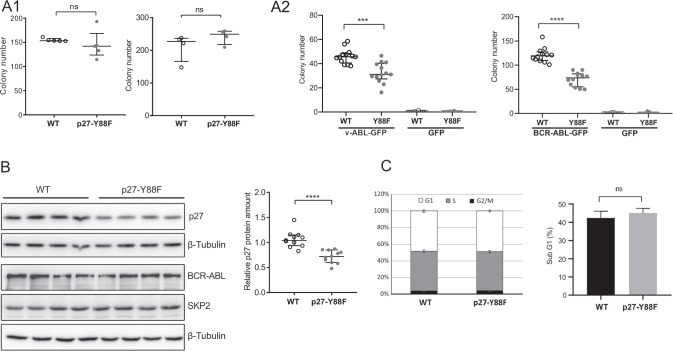
Reduced colony formation of ex vivo transformed p27-Y88F bone marrow cells. **A**1 Colony formation of wild type and p27-Y88F bone marrow progenitor B cells in methylcellulose containing 10 ng/ml rhIL-7 (left panel *n* = 5 males per genotype, 49–50 days of age, two independent experiments. Right panel *n* = 4 females per genotype, two independent experiments 47–50 days of age). **A**2 Left panel: v-ABL induced colony formation of wild type and p27-Y88F bone marrow cells in growth factor-free methylcellulose. Retroviral infection was performed in the presence of 10 ng/ml rhIL-7 (*n* = 13 mice per genotype, five independent experiments). Right panel: BCR::ABL1^p190^ induced colony formation of wild type and p27-Y88F bone marrow cells in growth factor-free methylcellulose. IL-7 was absent during the infection procedure (*n* = 12 mice per genotype, four independent experiments). **B** Representative immunoblots of BCR::ABL1^p190^ transformed wild type and p27-Y88F cells. After viral infection cells were maintained in B-cell culture medium and expanded for 9 days. Levels of p27, BCR::ABL1^p190^, SKP2 and β-tubulin as loading control are shown (left panel). Right panel: scatter dot blot represents quantification of p27 protein levels in BCR::ABL1^p190^ transformed wild type and p27-Y88F cells normalized to β-tubulin from three independent experiments (*n* = 11 mice per genotype). Protein levels were analyzed 9–11 days after infection. Normalized p27 protein levels in wild type cells were set to 1. **C** Representative graph of cell cycle phase distribution (left panel) and sub-G1 phase (right panel) of BCR::ABL1^p190^ transformed wild type and p27-Y88F cells cultured for 10 days. Percentage of cells in different cell cycle phases and sub-G1 phase were determined by flow cytometry. *n* = 3 mice per genotype. **A**1, **A**2 and **B** indicate the median and the interquartile range and statistical significance was determined by the Mann–Whitney *U* test (***p < 0.001, ****p < 0.0001).

To delineate if initial events of transformation contribute to accelerated leukemia progression in p27-Y88F mice, we expressed v-ABL and BCR::ABL1^p190^ in bone marrow cells derived from WT and p27-Y88F mice and tested their capacity to form colonies in growth factor-free semisolid media. As control, we first analyzed the ability of non-transformed p27-Y88F bone marrow cells to form progenitor B colonies in the presence of interleukin 7 (IL-7). A comparable number of colonies grew out from WT und p27-Y88F bone marrow cells isolated from females and males (Fig. 4A1), indicating that the potential of p27-Y88F bone marrow cells to generate progenitor B colonies is unaltered. B-cell identity of the colonies was confirmed by FACS (Supplementary Fig. 6). When primary bone marrow cells from WT and p27-Y88F mice were infected with v-ABL-pMSCV-IRES-GFP, significantly fewer p27-Y88F colonies grew out in growth factor-free semisolid media (average reduction of 29.5%) (Fig. 4A2 left panel), suggesting that p27-Y88F impairs initial transformation by v-ABL. We also tested the human orthologue BCR::ABL1^p190^, and performed the retroviral infection procedure in the absence or presence of IL-7, before seeding in growth factor-free semisolid media. Under both conditions, a significant reduction of p27-Y88F colony numbers was observed. The difference was even more pronounced in the absence of IL-7 pre-stimulation (41.2% reduction on average (Fig. 4A2 right panel) compared to 35.5% reduction on average in IL-7 pre-stimulated cells (Supplementary Fig. 7) suggesting that mitogenic and/or anti-apoptotic signaling by IL7 can support colony outgrowth in the presence of p27-Y88F. These findings indicate that p27-Y88F attenuates initial v-ABL and BCR::ABL1^p190^- induced B-cell transformation.

### p27-Y88F levels are significantly decreased in v-ABL and BCR::ABL1^p190^ transformed cells

v-ABL induced leukemia progression was accelerated in p27-Y88F mice, but initial transformation was diminished in the presence of p27-Y88F. We assumed that deregulation of p27-Y88F levels during transformation could contribute to accelerated tumor progression. Thus, selective pressure to proliferate in presence of p27-Y88F might induce downregulation of p27-Y88F levels. In fact, we observed a reproducible decrease of p27-Y88F levels in BCR::ABL1^p190^ transformed progenitor B cells expanded for 9 days in culture (Fig. 4B, average reduction of p27-Y88F levels by 32.6%) or in v-ABL transformed cells (Supplementary Fig. 8, average reduction of p27-Y88F levels by 39.3%). This indicates a strong selective pressure on v-ABL/BCR::ABL1^p190^ transformed cells to reduce p27 expression. Despite of the lower p27-Y88F levels, no significant differences in cell cycle phase distribution (Fig. 4C, left panel) or sub-G1 phase (Fig. 4C, right panel) were observed.

**Fig. 5:**
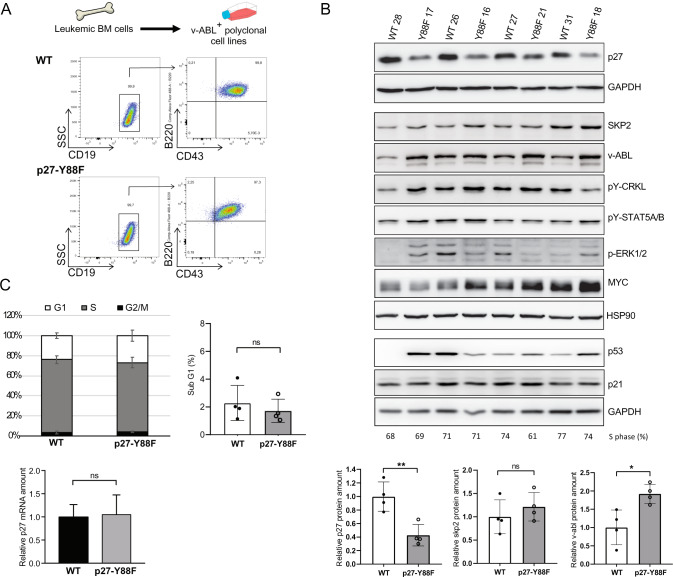
p27 protein levels are decreased in v-ABL^+^ p27-Y88F cell lines derived from leukemic mice. **A** Scheme of the generation of polyclonal v-ABL^+^ cell lines (upper panel) and representative FACS plots of B-cell marker staining (lower panels). Live cell population of v-ABL^+^ wild type and p27-Y88F cell lines were gated for CD19^+^ cells followed by B220 and CD43 as indicated by arrows. **B** Immunoblot analysis of wild type and p27-Y88F v-ABL^+^ cell lines generated from leukemic bone marrow cells shows protein levels of p27, SKP2, v-ABL, pY-CRKL, pY-STAT5A/B, p-ERK1/2, MYC, p53, p21 and GAPDH and HSP90 as loading controls. Densitometric analyses of p27 normalized to GAPDH, SKP2 and v-ABL levels normalized to HSP90 are shown below. Protein levels in wild type cells were set to 1. **C** Percentages of v-ABL^+^ wild type and p27-Y88F cell lines in G1, S and G2/M phase (left panel) and sub-G1 phase (right panel) as determined by flow cytometry. Lower panel displays p27 mRNA levels determined by qPCR in wild type and p27-Y88F v-ABL^+^ cell lines. *n* = 4 per genotype. Cell lines correspond to those used in **B**. One representative experiment out of three is shown. Data represent mean ± SD. (*p < 0.05, **p < 0.01, ***p < 0.001).

To investigate if the downregulation of p27-Y88F protein levels also occurs in leukemia-derived cells, we immortalized bone marrow cells from v-ABL diseased mice. These v-ABL^+^ polyclonal cell populations expressed the characteristic surface markers B220, CD19 and CD43 [29] (Fig. 5A and Supplementary Fig. 9). Their genotypes were confirmed by PCR (Supplementary Fig. 10). Immunoblot analysis revealed a pronounced decrease of p27 levels in v-ABL^+^ p27-Y88F cells compared to v-ABL^+^ WT cells (Fig. 5B). With an average reduction by 57%, this decrease was even more pronounced than in ex vivo transformed p27-Y88F cells. Expression levels of D-type cyclins, cyclin A, CDK2, CDK4 and p21 varied, but were not selectively different between WT and p27-Y88F v-ABL^+^ cells (Supplementary Fig. 11). Increased v-ABL protein levels in v-ABL^+^ cells suggest that enhanced v-ABL expression may be required to promote proliferation and/or transformation of p27-Y88F cells in this model (Fig. 5B). Enhanced phosphorylation of the direct v-ABL/BCR::ABL1^p190^ substrate CRKL was detected in three out of four p27-Y88F cell lines (Fig. 5B). Phosphorylation of the v-ABL downstream targets STAT5A/B and ERK1/2 varied strongly within clones and expression of MYC and p53 showed a stochastic pattern (Fig. 5B). p27-Y88F v-ABL^+^ cells showed no specific deregulation of SKP2 protein (Fig. 5B) or changes in p27 mRNA levels compared to the respective WT cells (Fig. 5C lower panel) ruling out that the deregulation of p27 protein amount in v-ABL^+^ cells occurs at the transcriptional level or by Skp2 regulation.

Cell cycle phase distribution and frequency of apoptotic sub-G1 cells remained unaltered in v-ABL^+^ p27-Y88F cells (Fig. 5C upper panels and Supplementary Fig. 12), indicating that the low p27-Y88F levels do not alter apoptosis. The similar cell cycle profile excludes cell cycle position effects. Together, the analysis of v-ABL transformed leukemia cells revealed a strong and consistent downregulation of p27-Y88F levels, which could indicate a non-redundant requirement for decreased p27-Y88F levels to permit v-ABL-induced cell proliferation and transformation.

### The transformative potential of p27-Y88F v-ABL^+^ cell lines is enhanced

**Fig. 6:**
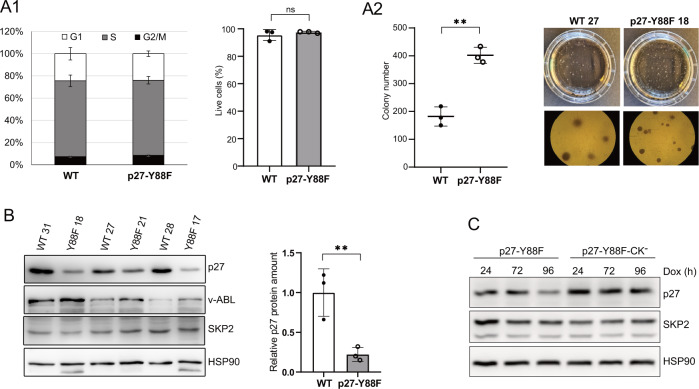
Colony formation capacity of established p27-Y88F v-ABL^+^ cell lines is increased and downregulation of p27-Y88F depends on cyclin-CDK binding. **A**1 Left graph represents cell cycle phase distribution of v-ABL^+^ wild type and p27-Y88F cells as determined by flow cytometry after propidium iodide and anti-BrdU staining. Right graph indicates percentages of live cells identified by flow cytometry as cells staining negative for Annexin V and DAPI. **A**2 Colony formation assay of wild type and p27-Y88F v-ABL^+^ cells. Left panel displays colony numbers of one representative experiment out of two. Right panel shows photographs of a representative wild type and p27-Y88F colony formation assay. **B** Cells isolated from colonies were subjected to western blot. p27, v-ABL and SKP2 protein levels were analyzed. HSP90 was used as loading control. Right panel presents densitometric analysis of p27 protein levels normalized to HSP90. Data represent mean ± SD. (**p < 0.01). **C** p27-Y88F or p27-Y88F-CK^−^ expression was induced with 1 μg/ml doxycycline in HeLa Flp-In cells for 24, 72 and 96 h and p27, SKP2 and HSP90 protein levels were analyzed by western blot. One representative experiment out of three is shown.

Since p27-Y88F levels were importantly decreased in v-ABL^+^ cell lines and low p27 levels predispose to tumorigenesis [1, 19], we speculated that cells with severely reduced p27-Y88F levels cause accelerated v-ABL/BCR::ABL1^p190^ mediated transformation. Since leukemia-derived v-ABL^+^ cells could serve as a model to investigate advanced stages of transformation and the transformative potential can be monitored by colony formation assays [30], we tested their potential to form colonies in growth factor-free methylcellulose. Investigating cell lines with similar cell cycle profiles and degree of apoptosis (Fig. 6A1), p27-Y88F cell lines gave rise to significantly more colonies and showed a tendency to form smaller, but denser colonies than the WT cell lines (Fig. 6A2). In cells isolated from these colonies, a dramatically reduced amount of p27-Y88F protein was expressed compared to WT p27 (Fig. 6B). SKP2 protein levels remained largely unaltered and v-ABL levels varied strongly among individual cell lines (Fig. 6B). These results reveal an increased transformative potential of v-ABL^+^ p27-Y88F cell lines, which is likely due to the pronounced decrease of p27-Y88F levels.

In order to better characterize the molecular mechanism leading to decreased p27 levels in p27-Y88 expressing cells, we speculated that the robust CDK inhibition by p27-Y88F might exert a pressure on cells to lower p27 levels. To test if the reduction of p27 levels depends on its CDK-binding and -inhibition, we inducibly expressed p27-Y88F and a cyclin/CDK-binding deficient p27-Y88F variant (p27-Y88F-CK^−^) in HeLa Flp-In cells. Following induction, p27-Y88F levels were decreased and remained importantly reduced throughout doxycycline treatment compared to p27-Y88F-CK^-^ levels at the respective time points (Fig. 6C, reduction by 39% and 51% at 24 and 96 h respectively). This demonstrates that CDK binding is a prerequisite for the downregulation of p27-Y88F expression.

Together, these data support the model that during the initial phase of transformation, the amount of p27-Y88F is sufficient to prevent oncogene-induced proliferation. However, cells emerging with very low p27-Y88F levels would enable cell proliferation and transformation by ABL oncogenes resulting in accelerated leukemia progression.

## Discussion

The phosphorylation of p27 on Y88 plays a central role in the assembly of active cyclin D/CDK4 complexes, impairs cyclins A, E/CDK2 inhibition and enhances p27 destabilization [1]. Blocking p27-Y88 phosphorylation by Brk-SH3 peptide efficiently inhibited CDK4 and CDK2 activity and arrested proliferation of breast cancer cells in a xenograft model [31]. A correlation of palbociclib responsiveness and the p27-Y88 phosphorylation status was detected in breast cancer explant cultures [32]. Oncogenic tyrosine kinases such as BCR::ABL1, SRC family kinases, BRK, JAK2-V617F and FLT3-ITD share p27 as a common substrate and phosphorylate Y88 [7–11]. To directly study the contribution of Y88-phosphorylation in leukemogenesis, we have generated the p27-Y88F mouse model. We speculated that the p27-Y88F protein would act as a robust CDK inhibitor and hinder cell proliferation and transformation due to its resistance to tyrosine kinase-induced p27 inactivation by Y88-phosphorylation. Surprisingly, we observed the opposite, namely a faster progression of v-ABL induced B-cell leukemia/lymphoma in p27-Y88F animals. This was likely caused by downregulation of p27-Y88F expression.

p27 controls cell proliferation in multiple mouse organs, which is reflected by increased body weight and organ size of p27^+/−^ and p27^−/−^ knock out mice [33–35]. Lower body weight of young p27-Y88F mice was associated with reduced cell counts in hematopoietic organs, which is consistent with the stronger CDK inhibition by p27-Y88F. It is conceivable that the recovery of body weight observed upon maturation is due to the compensatory downregulation of p27-Y88F protein levels.

The reduced level of p27-Y88F protein observed in cells from various organs of knock-in mice underscores the central role of p27-Y88 phosphorylation in normal growth and development. p27-Y88F levels must suffice to restrict proliferation upon initial transformation by v-ABL and BCR::ABL1^p190^, as the outgrowth of p27-Y88F colonies was diminished when cells were immediately subjected to colony formation assays after oncogene expression. Levels of p27-Y88F directly after oncogene expression could not be investigated due to the limited amount of cells. However, modestly reduced expression of p27-Y88F in these cells is conceivable since p27-Y88F levels were also decreased in non-transformed hematopoietic cells.

In contrast, v-ABL^+^ cell lines derived from diseased p27-Y88F mice with accelerated disease progression were characterized by a severe reduction of p27-Y88F levels and an increased colony formation potential, suggesting that in later stages of transformation, p27-Y88F levels decrease below the critical threshold required for efficient CDK inhibition resulting in enhanced leukemogenesis. The downregulation of p27-Y88F levels requires its ability to inhibit cyclin/CDK activity, since expression of the p27-Y88F mutant deficient in cyclin/CDK binding was not decreased. This supports the hypothesis that the pronounced downregulation of p27-Y88F levels is caused by its ability to strongly inhibit cyclin/CDK activity. The subsequently faster disease progression in p27-Y88F animals is consistent with earlier observations that reduced p27 levels in heterozygous or homozygous p27 knock-out mice predispose to aggressive tumor development after exposure to carcinogens or expression of oncogenes including BCR::ABL1^p210^ [19, 36, 37].

The v-ABL target MYC is a pleiotropic transcription factor that controls proliferation and survival of normal and malignant cells and regulates p27, CDK2, cyclins E and A and Skp2 [38]. MYC activation occurs more frequently in Moloney murine leukemia virus induced lymphomas in p27 knock-out than in WT tumors [39]. While high MYC expression was observed in several v-ABL transformed cell lines and is likely to contribute to transformation, increased MYC levels did not correlate with the p27 or p27-Y88F status (Fig. 5B). Similarly, levels of D-type cyclins, which can be overexpressed in cancer cells, and p53 varied among cell lines but again without a significant preference for p27 or p27-Y88F cells (Supplementary Fig. 11). The activation of different oncogenic pathways during individual tumor evolution seems similar in WT and p27-Y88F leukemia-derived cells with no selective preference for p27-Y88F. The reduced expression of p27-Y88F in mouse tissues and in all leukemia-derived cell lines appears non-redundant and uncovers the downregulation of p27-Y88F levels as essential mechanism to overcome the strong and constant CDK inhibition by constitutive p27-Y88F expression. The requirement of cyclin/CDK binding of p27-Y88F for its downregulation underpins this finding.

All human and mouse Cip/Kip proteins share an inhibitory 3_10_-helix with a conserved tyrosine residue in a position homologous to Y88 of p27, which can be phosphorylated [40]. One might therefore speculate that p21^Cip1^ or p57^Kip2^ compensates for some essential functions of Y88 phosphorylated p27. p57^Kip2^ was not detectable in v-ABL transformed cell lines (data not shown). p21^Cip1^ was expressed in v-ABL^+^ cell lines and in thymocytes, but protein levels were not altered between WT and p27-Y88F (Fig. 5B and Supplementary Fig. 3). The single tyrosine residue of p21, tyrosine-77, is a substrate of v-ABL in vitro [40]. However, in contrast to Y88-phosphorylated p27, Y77-phosphorylated p21 is still able to completely inhibit cyclin A/CDK2 [40] and Y77-phosphorylation of p21 cannot activate cyclin D/CDK4 [15].

The mechanism uncovered here for the posttranscriptional downregulation of p27-Y88F levels in knock-in cells is independent of SKP2 deregulation and involves cyclin/CDK binding. This mechanism may promote tumor growth during oncogene transformation when p27 does not become phosphorylated on Y88 as part of the oncogenic process.

As many human tumors are characterized by low p27 expression [1], and small molecule CDK4,6 inhibitors are used in cancer therapy, pathway(s) lowering p27 levels upon sustained CDK inhibition might also become activated in human malignancies, contribute to chemoresistance and the outgrowth of more aggressive tumors upon relapse. It will therefore be important to determine the precise molecular mechanism that reduces p27-Y88F levels.

## Supplementary information

Supplemental Material
