# Supplementary material for: Inability to phosphorylate Y88 of p27Kip1 enforces reduced p27 protein levels and accelerates leukemia progression
Source: Leukemia. 2022 May 21;36(7):1916–25. doi: 10.1038/s41375-022-01598-x (PMC9252907; doi:10.1038/s41375-022-01598-x)
Supplement: Supplementary file 1 — Supplemental Material [file 41375_2022_1598_MOESM1_ESM.pptx]

## Slide 1
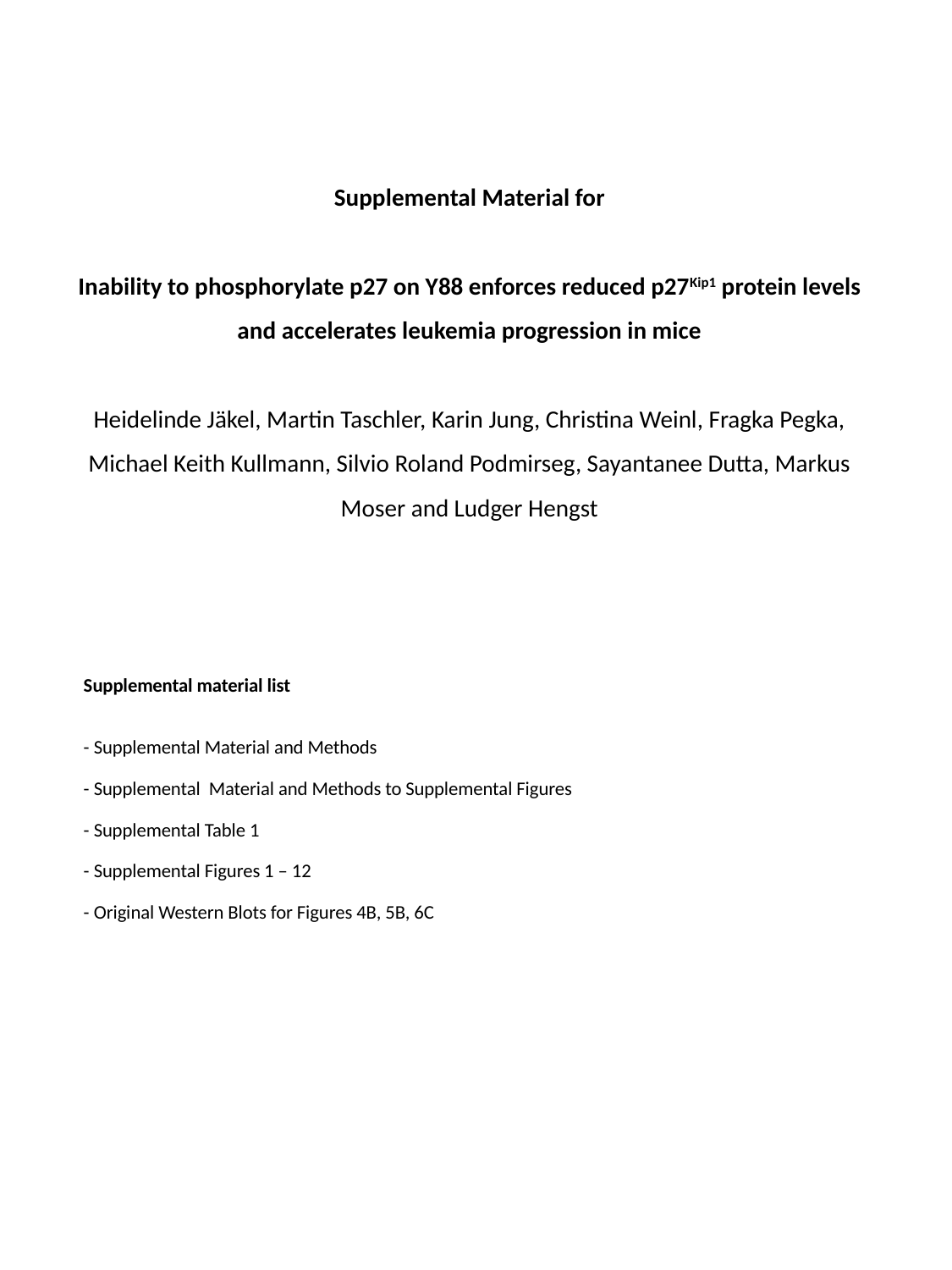

# Supplemental Material for Inability to phosphorylate p27 on Y88 enforces reduced p27Kip1 protein levels and accelerates leukemia progression in mice Heidelinde Jäkel, Martin Taschler, Karin Jung, Christina Weinl, Fragka Pegka, Michael Keith Kullmann, Silvio Roland Podmirseg, Sayantanee Dutta, Markus Moser and Ludger Hengst
Supplemental material list
- Supplemental Material and Methods
- Supplemental Material and Methods to Supplemental Figures
- Supplemental Table 1
- Supplemental Figures 1 – 12
- Original Western Blots for Figures 4B, 5B, 6C

## Slide 2
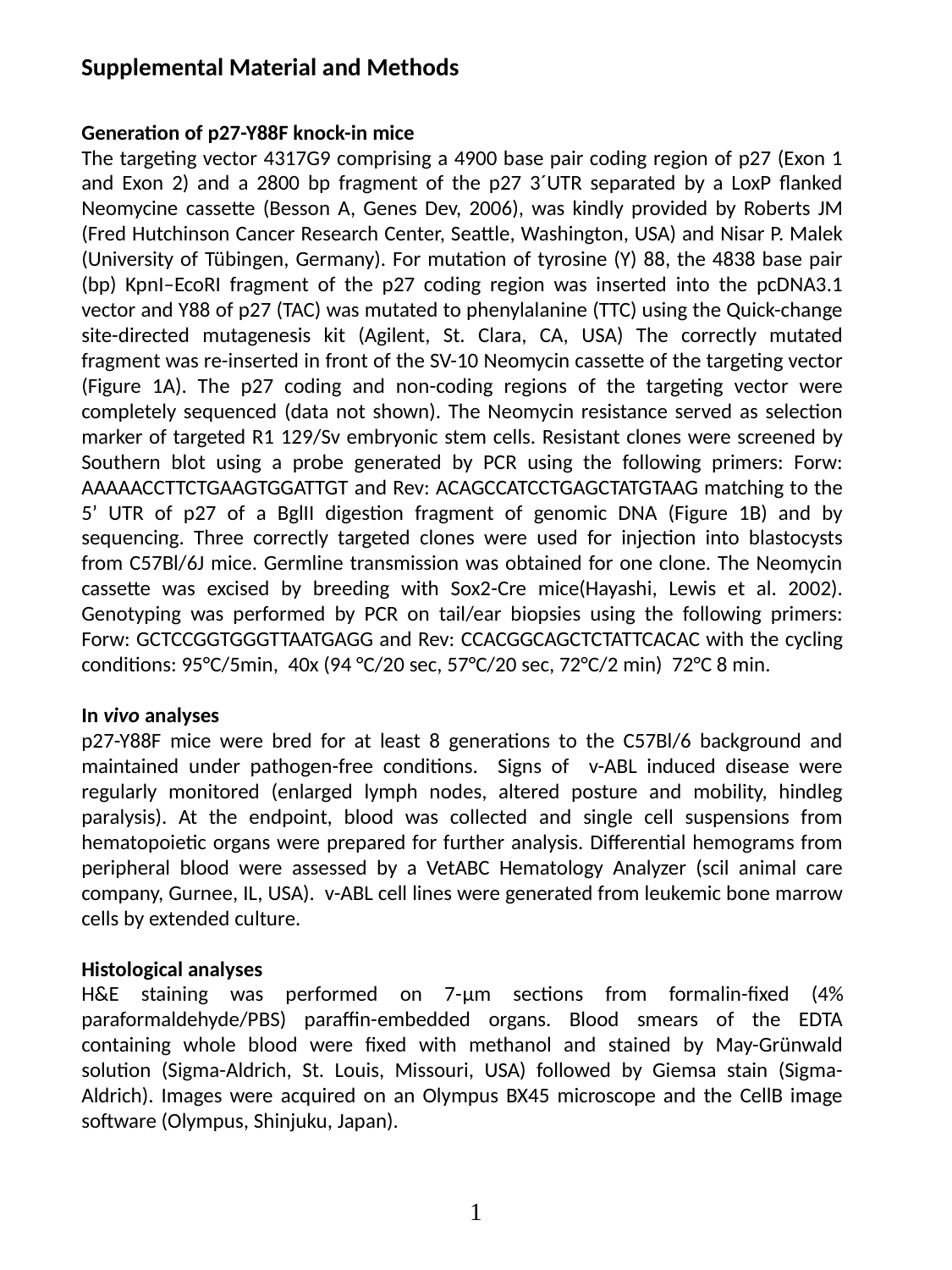

Supplemental Material and Methods
Generation of p27-Y88F knock-in mice
The targeting vector 4317G9 comprising a 4900 base pair coding region of p27 (Exon 1 and Exon 2) and a 2800 bp fragment of the p27 3´UTR separated by a LoxP flanked Neomycine cassette (Besson A, Genes Dev, 2006), was kindly provided by Roberts JM (Fred Hutchinson Cancer Research Center, Seattle, Washington, USA) and Nisar P. Malek (University of Tübingen, Germany). For mutation of tyrosine (Y) 88, the 4838 base pair (bp) KpnI–EcoRI fragment of the p27 coding region was inserted into the pcDNA3.1 vector and Y88 of p27 (TAC) was mutated to phenylalanine (TTC) using the Quick-change site-directed mutagenesis kit (Agilent, St. Clara, CA, USA) The correctly mutated fragment was re-inserted in front of the SV-10 Neomycin cassette of the targeting vector (Figure 1A). The p27 coding and non-coding regions of the targeting vector were completely sequenced (data not shown). The Neomycin resistance served as selection marker of targeted R1 129/Sv embryonic stem cells. Resistant clones were screened by Southern blot using a probe generated by PCR using the following primers: Forw: AAAAACCTTCTGAAGTGGATTGT and Rev: ACAGCCATCCTGAGCTATGTAAG matching to the 5’ UTR of p27 of a BglII digestion fragment of genomic DNA (Figure 1B) and by sequencing. Three correctly targeted clones were used for injection into blastocysts from C57Bl/6J mice. Germline transmission was obtained for one clone. The Neomycin cassette was excised by breeding with Sox2-Cre mice(Hayashi, Lewis et al. 2002). Genotyping was performed by PCR on tail/ear biopsies using the following primers: Forw: GCTCCGGTGGGTTAATGAGG and Rev: CCACGGCAGCTCTATTCACAC with the cycling conditions: 95°C/5min, 40x (94 °C/20 sec, 57°C/20 sec, 72°C/2 min) 72°C 8 min.
In vivo analyses
p27-Y88F mice were bred for at least 8 generations to the C57Bl/6 background and maintained under pathogen-free conditions. Signs of v-ABL induced disease were regularly monitored (enlarged lymph nodes, altered posture and mobility, hindleg paralysis). At the endpoint, blood was collected and single cell suspensions from hematopoietic organs were prepared for further analysis. Differential hemograms from peripheral blood were assessed by a VetABC Hematology Analyzer (scil animal care company, Gurnee, IL, USA). v-ABL cell lines were generated from leukemic bone marrow cells by extended culture.
Histological analyses
H&E staining was performed on 7-μm sections from formalin-fixed (4% paraformaldehyde/PBS) paraffin-embedded organs. Blood smears of the EDTA containing whole blood were fixed with methanol and stained by May-Grünwald solution (Sigma-Aldrich, St. Louis, Missouri, USA) followed by Giemsa stain (Sigma-Aldrich). Images were acquired on an Olympus BX45 microscope and the CellB image software (Olympus, Shinjuku, Japan).
1

## Slide 3
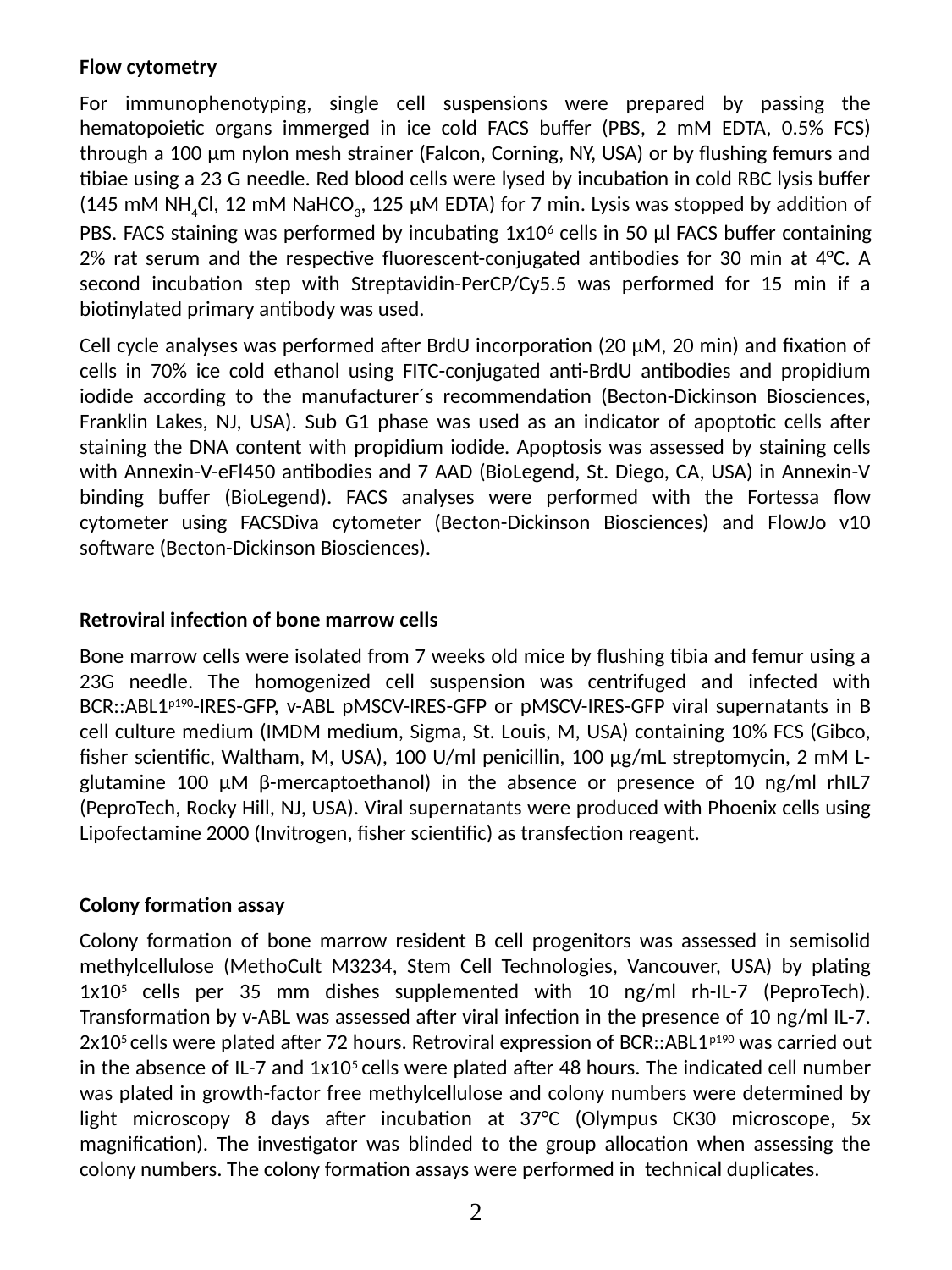

Flow cytometry
For immunophenotyping, single cell suspensions were prepared by passing the hematopoietic organs immerged in ice cold FACS buffer (PBS, 2 mM EDTA, 0.5% FCS) through a 100 μm nylon mesh strainer (Falcon, Corning, NY, USA) or by flushing femurs and tibiae using a 23 G needle. Red blood cells were lysed by incubation in cold RBC lysis buffer (145 mM NH4Cl, 12 mM NaHCO3, 125 μM EDTA) for 7 min. Lysis was stopped by addition of PBS. FACS staining was performed by incubating 1x10^6^ cells in 50 μl FACS buffer containing 2% rat serum and the respective fluorescent-conjugated antibodies for 30 min at 4°C. A second incubation step with Streptavidin-PerCP/Cy5.5 was performed for 15 min if a biotinylated primary antibody was used.
Cell cycle analyses was performed after BrdU incorporation (20 μM, 20 min) and fixation of cells in 70% ice cold ethanol using FITC-conjugated anti-BrdU antibodies and propidium iodide according to the manufacturer´s recommendation (Becton-Dickinson Biosciences, Franklin Lakes, NJ, USA). Sub G1 phase was used as an indicator of apoptotic cells after staining the DNA content with propidium iodide. Apoptosis was assessed by staining cells with Annexin-V-eFl450 antibodies and 7 AAD (BioLegend, St. Diego, CA, USA) in Annexin-V binding buffer (BioLegend). FACS analyses were performed with the Fortessa flow cytometer using FACSDiva cytometer (Becton-Dickinson Biosciences) and FlowJo v10 software (Becton-Dickinson Biosciences).
Retroviral infection of bone marrow cells
Bone marrow cells were isolated from 7 weeks old mice by flushing tibia and femur using a 23G needle. The homogenized cell suspension was centrifuged and infected with BCR::ABL1p190-IRES-GFP, v-ABL pMSCV-IRES-GFP or pMSCV-IRES-GFP viral supernatants in B cell culture medium (IMDM medium, Sigma, St. Louis, M, USA) containing 10% FCS (Gibco, fisher scientific, Waltham, M, USA), 100 U/ml penicillin, 100 μg/mL streptomycin, 2 mM L-glutamine 100 µM β-mercaptoethanol) in the absence or presence of 10 ng/ml rhIL7 (PeproTech, Rocky Hill, NJ, USA). Viral supernatants were produced with Phoenix cells using Lipofectamine 2000 (Invitrogen, fisher scientific) as transfection reagent.
Colony formation assay
Colony formation of bone marrow resident B cell progenitors was assessed in semisolid methylcellulose (MethoCult M3234, Stem Cell Technologies, Vancouver, USA) by plating 1x10^5^ cells per 35 mm dishes supplemented with 10 ng/ml rh-IL-7 (PeproTech). Transformation by v-ABL was assessed after viral infection in the presence of 10 ng/ml IL-7. 2x10^5^ cells were plated after 72 hours. Retroviral expression of BCR::ABL1p190 was carried out in the absence of IL-7 and 1x10^5^ cells were plated after 48 hours. The indicated cell number was plated in growth-factor free methylcellulose and colony numbers were determined by light microscopy 8 days after incubation at 37°C (Olympus CK30 microscope, 5x magnification). The investigator was blinded to the group allocation when assessing the colony numbers. The colony formation assays were performed in technical duplicates.
2

## Slide 4
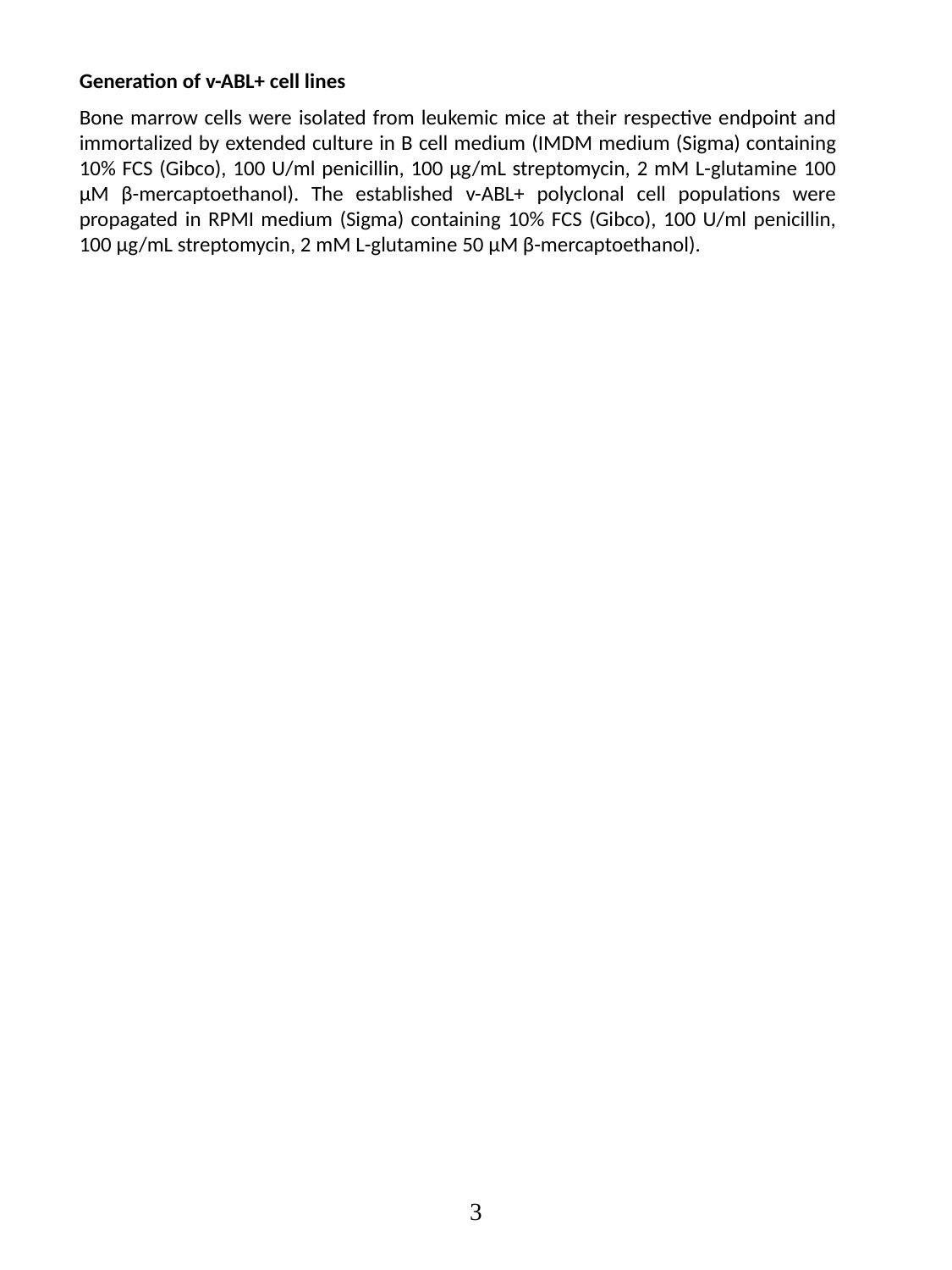

Generation of v-ABL+ cell lines
Bone marrow cells were isolated from leukemic mice at their respective endpoint and immortalized by extended culture in B cell medium (IMDM medium (Sigma) containing 10% FCS (Gibco), 100 U/ml penicillin, 100 μg/mL streptomycin, 2 mM L-glutamine 100 µM β-mercaptoethanol). The established v-ABL+ polyclonal cell populations were propagated in RPMI medium (Sigma) containing 10% FCS (Gibco), 100 U/ml penicillin, 100 μg/mL streptomycin, 2 mM L-glutamine 50 µM β-mercaptoethanol).
3

## Slide 5
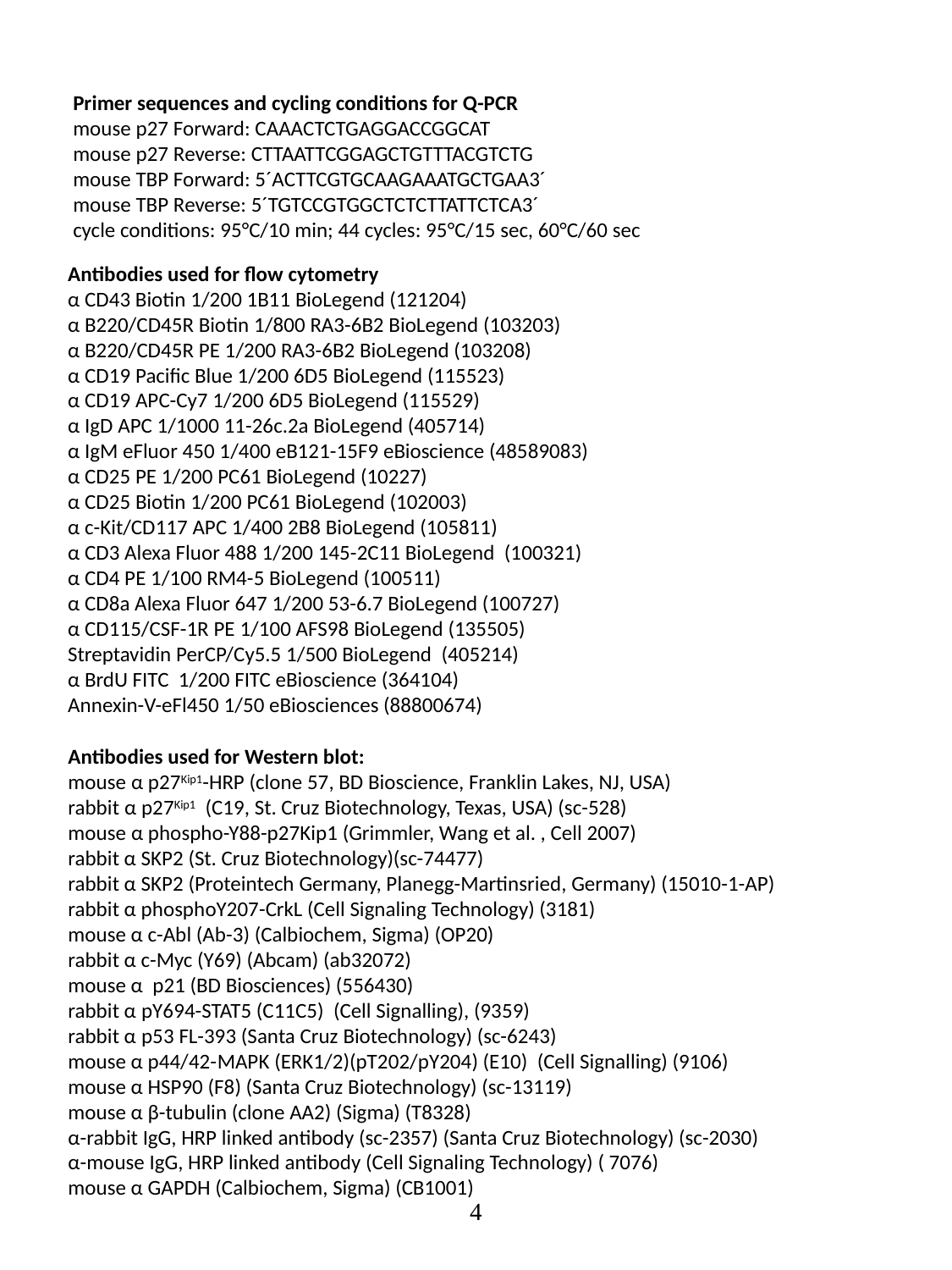

Primer sequences and cycling conditions for Q-PCR
mouse p27 Forward: CAAACTCTGAGGACCGGCAT
mouse p27 Reverse: CTTAATTCGGAGCTGTTTACGTCTG
mouse TBP Forward: 5´ACTTCGTGCAAGAAATGCTGAA3´
mouse TBP Reverse: 5´TGTCCGTGGCTCTCTTATTCTCA3´
cycle conditions: 95°C/10 min; 44 cycles: 95°C/15 sec, 60°C/60 sec
Antibodies used for flow cytometry
α CD43 Biotin 1/200 1B11 BioLegend (121204)
α B220/CD45R Biotin 1/800 RA3-6B2 BioLegend (103203)
α B220/CD45R PE 1/200 RA3-6B2 BioLegend (103208)
α CD19 Pacific Blue 1/200 6D5 BioLegend (115523)
α CD19 APC-Cy7 1/200 6D5 BioLegend (115529)
α IgD APC 1/1000 11-26c.2a BioLegend (405714)
α IgM eFluor 450 1/400 eB121-15F9 eBioscience (48589083)
α CD25 PE 1/200 PC61 BioLegend (10227)
α CD25 Biotin 1/200 PC61 BioLegend (102003)
α c-Kit/CD117 APC 1/400 2B8 BioLegend (105811)
α CD3 Alexa Fluor 488 1/200 145-2C11 BioLegend (100321)
α CD4 PE 1/100 RM4-5 BioLegend (100511)
α CD8a Alexa Fluor 647 1/200 53-6.7 BioLegend (100727)
α CD115/CSF-1R PE 1/100 AFS98 BioLegend (135505)
Streptavidin PerCP/Cy5.5 1/500 BioLegend (405214)
α BrdU FITC 1/200 FITC eBioscience (364104)
Annexin-V-eFl450 1/50 eBiosciences (88800674)
Antibodies used for Western blot:
mouse α p27Kip1-HRP (clone 57, BD Bioscience, Franklin Lakes, NJ, USA)
rabbit α p27Kip1 (C19, St. Cruz Biotechnology, Texas, USA) (sc-528)
mouse α phospho-Y88-p27Kip1 (Grimmler, Wang et al. , Cell 2007)
rabbit α SKP2 (St. Cruz Biotechnology)(sc-74477)
rabbit α SKP2 (Proteintech Germany, Planegg-Martinsried, Germany) (15010-1-AP)
rabbit α phosphoY207-CrkL (Cell Signaling Technology) (3181)
mouse α c-Abl (Ab-3) (Calbiochem, Sigma) (OP20)
rabbit α c-Myc (Y69) (Abcam) (ab32072)
mouse α p21 (BD Biosciences) (556430)
rabbit α pY694-STAT5 (C11C5) (Cell Signalling), (9359)
rabbit α p53 FL-393 (Santa Cruz Biotechnology) (sc-6243)
mouse α p44/42-MAPK (ERK1/2)(pT202/pY204) (E10) (Cell Signalling) (9106)
mouse α HSP90 (F8) (Santa Cruz Biotechnology) (sc-13119)
mouse α β-tubulin (clone AA2) (Sigma) (T8328)
α-rabbit IgG, HRP linked antibody (sc-2357) (Santa Cruz Biotechnology) (sc-2030)
α-mouse IgG, HRP linked antibody (Cell Signaling Technology) ( 7076)
mouse α GAPDH (Calbiochem, Sigma) (CB1001)
4

## Slide 6
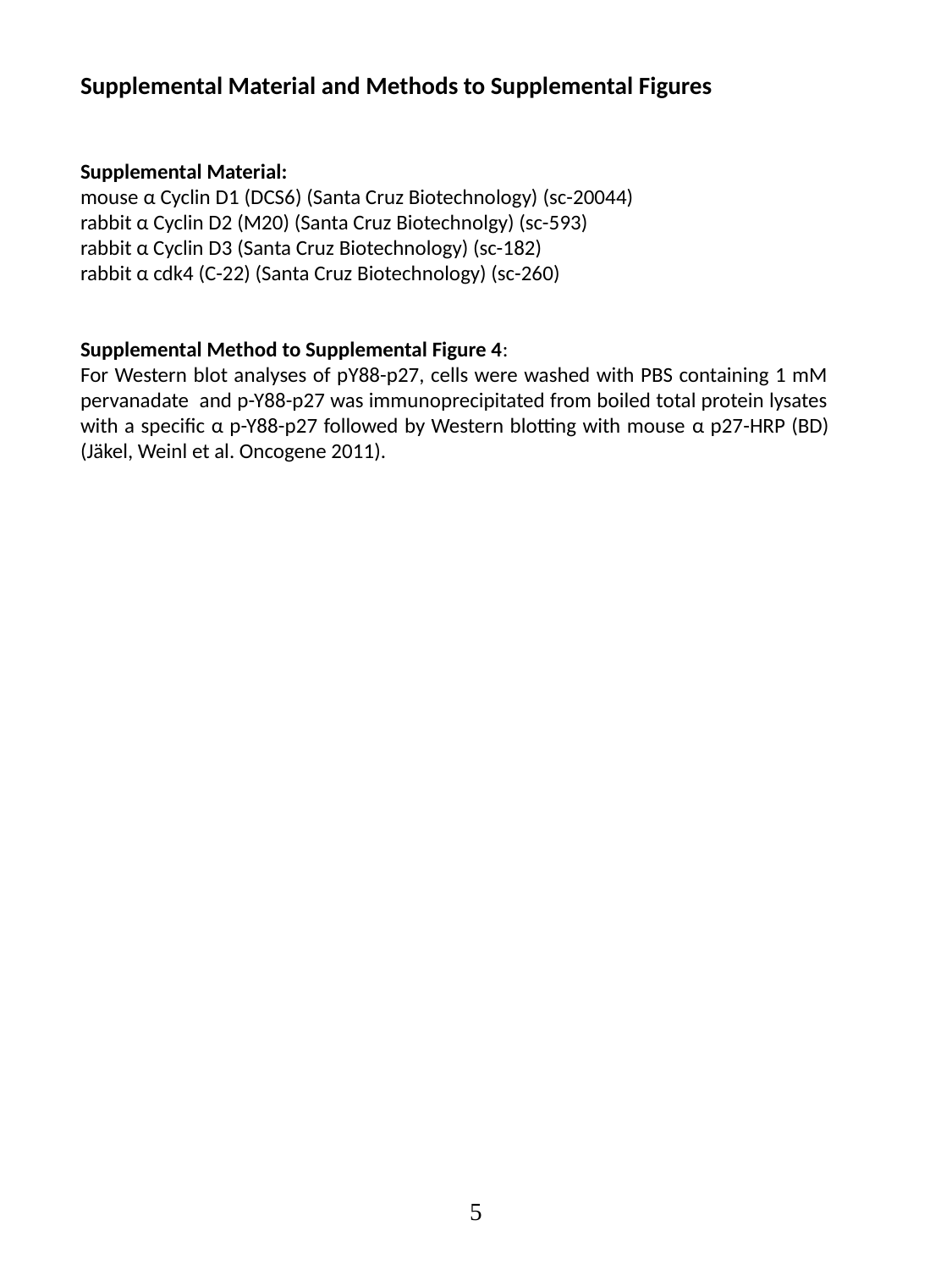

Supplemental Material and Methods to Supplemental Figures
Supplemental Material:
mouse α Cyclin D1 (DCS6) (Santa Cruz Biotechnology) (sc-20044)
rabbit α Cyclin D2 (M20) (Santa Cruz Biotechnolgy) (sc-593)
rabbit α Cyclin D3 (Santa Cruz Biotechnology) (sc-182)
rabbit α cdk4 (C-22) (Santa Cruz Biotechnology) (sc-260)
Supplemental Method to Supplemental Figure 4:
For Western blot analyses of pY88-p27, cells were washed with PBS containing 1 mM pervanadate and p-Y88-p27 was immunoprecipitated from boiled total protein lysates with a specific α p-Y88-p27 followed by Western blotting with mouse α p27-HRP (BD) (Jäkel, Weinl et al. Oncogene 2011).
5

## Slide 7
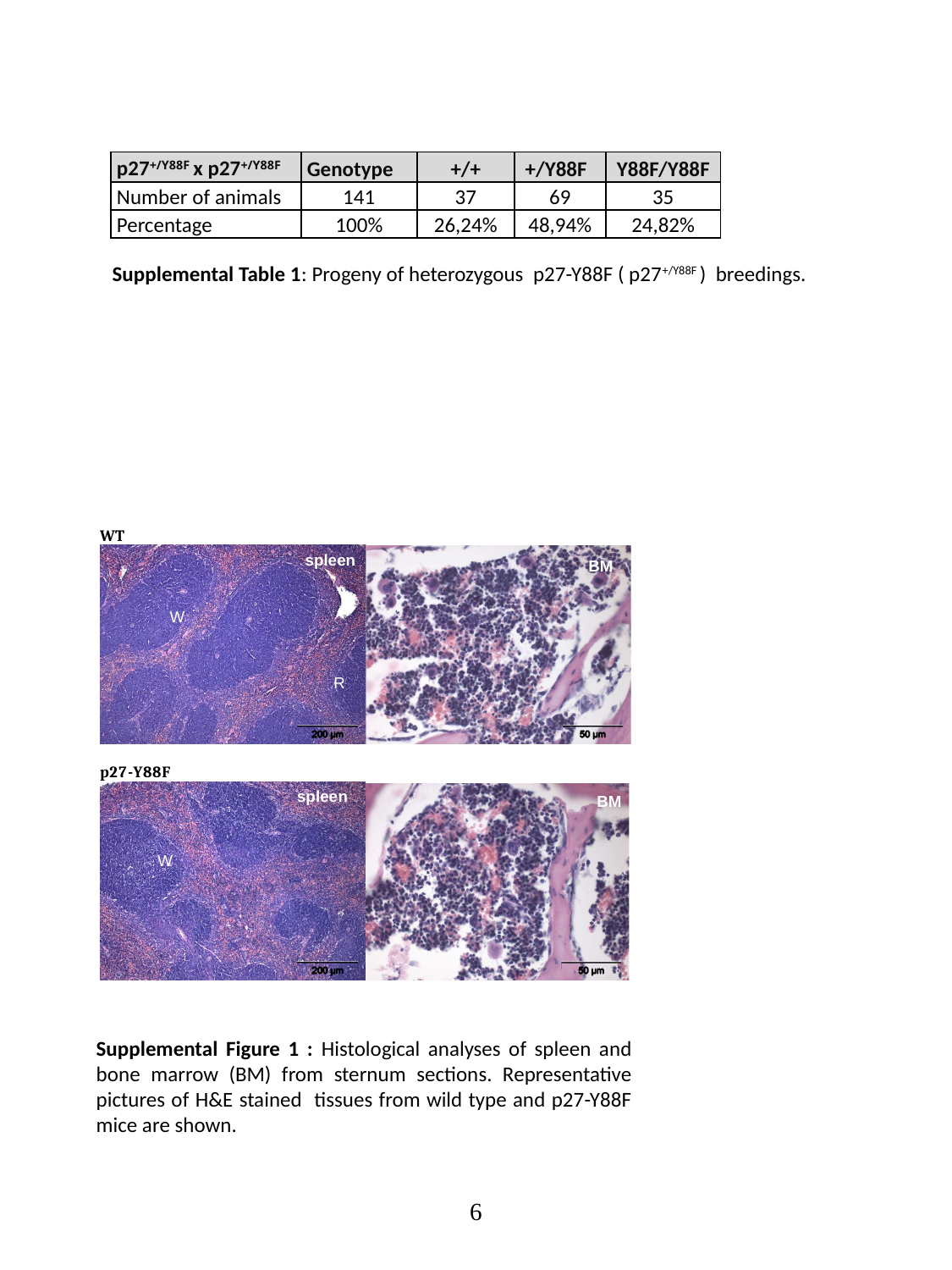

| p27+/Y88F x p27+/Y88F | Genotype | +/+ | +/Y88F | Y88F/Y88F |
| --- | --- | --- | --- | --- |
| Number of animals | 141 | 37 | 69 | 35 |
| Percentage | 100% | 26,24% | 48,94% | 24,82% |
Supplemental Table 1: Progeny of heterozygous p27-Y88F ( p27+/Y88F ) breedings.
Supplemental Figure 1 : Histological analyses of spleen and bone marrow (BM) from sternum sections. Representative pictures of H&E stained tissues from wild type and p27-Y88F mice are shown.
6

## Slide 8
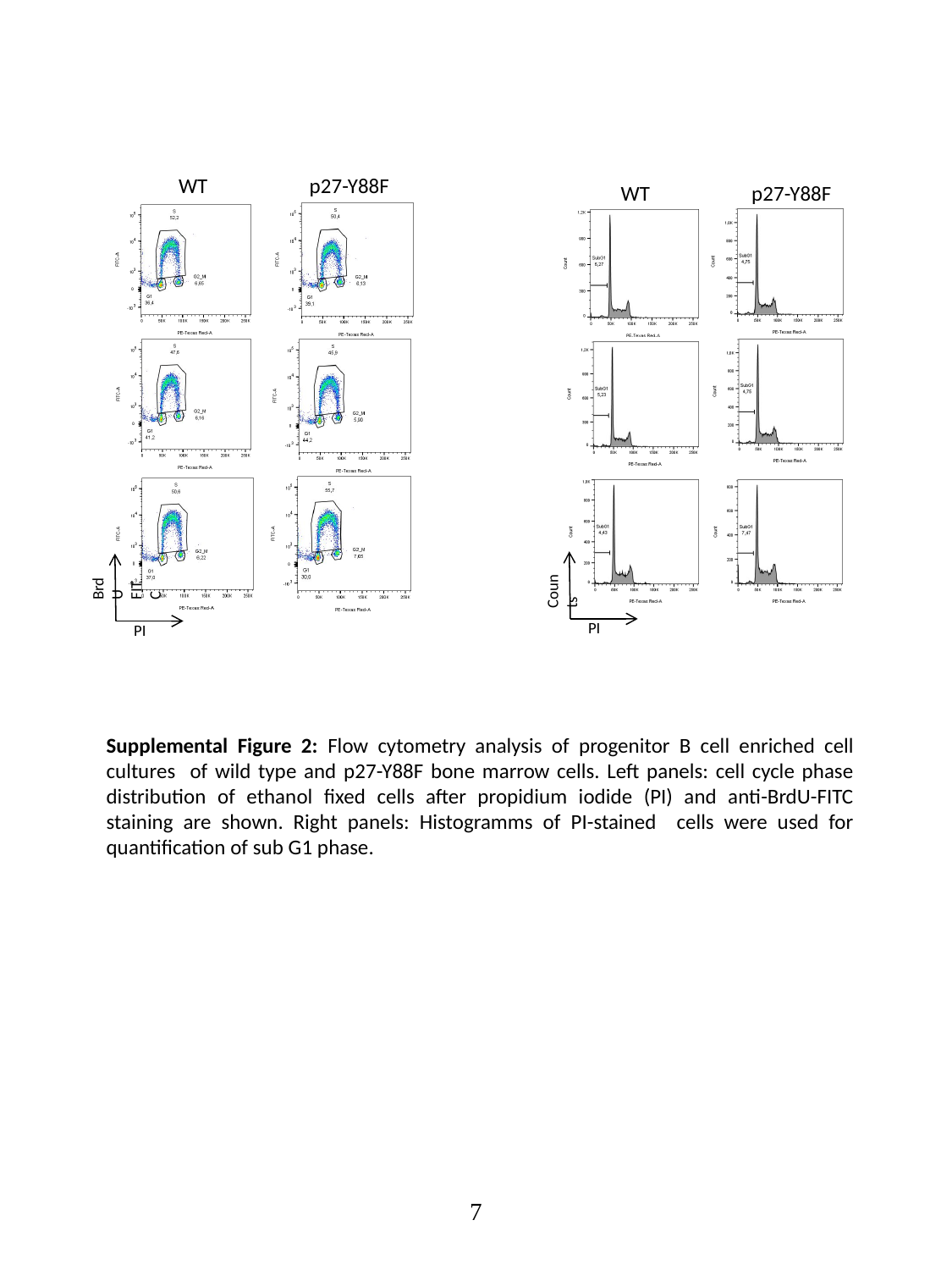

WT p27-Y88F
BrdU FITC
PI
WT p27-Y88F
Counts
PI
Supplemental Figure 2: Flow cytometry analysis of progenitor B cell enriched cell cultures of wild type and p27-Y88F bone marrow cells. Left panels: cell cycle phase distribution of ethanol fixed cells after propidium iodide (PI) and anti-BrdU-FITC staining are shown. Right panels: Histogramms of PI-stained cells were used for quantification of sub G1 phase.
7

## Slide 9
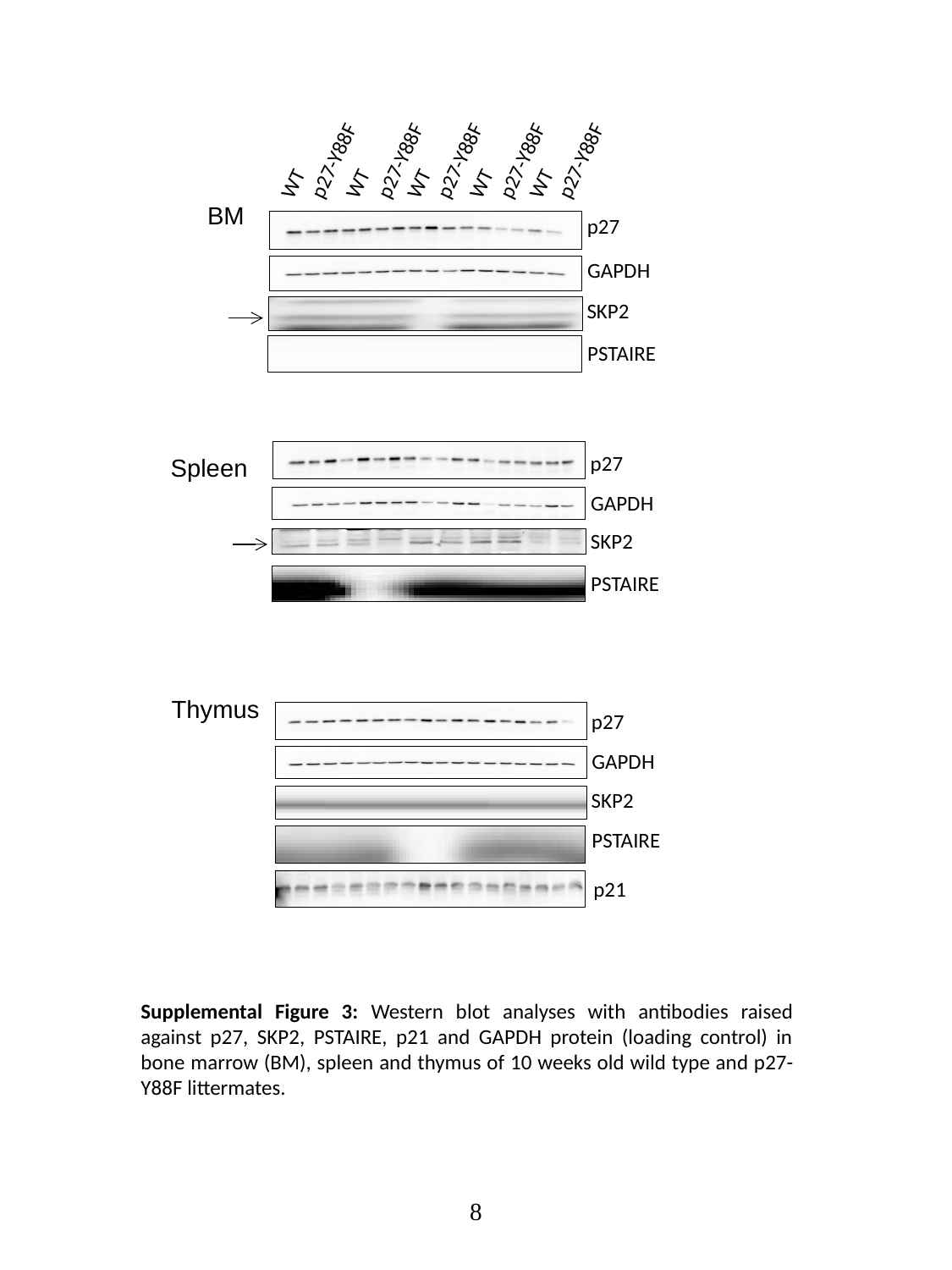

WT
p27-Y88F
WT
p27-Y88F
WT
p27-Y88F
WT
p27-Y88F
WT
p27-Y88F
BM
p27
GAPDH
SKP2
PSTAIRE
p27
Spleen
GAPDH
SKP2
PSTAIRE
Thymus
p27
GAPDH
SKP2
PSTAIRE
p21
Supplemental Figure 3: Western blot analyses with antibodies raised against p27, SKP2, PSTAIRE, p21 and GAPDH protein (loading control) in bone marrow (BM), spleen and thymus of 10 weeks old wild type and p27-Y88F littermates.
8

## Slide 10
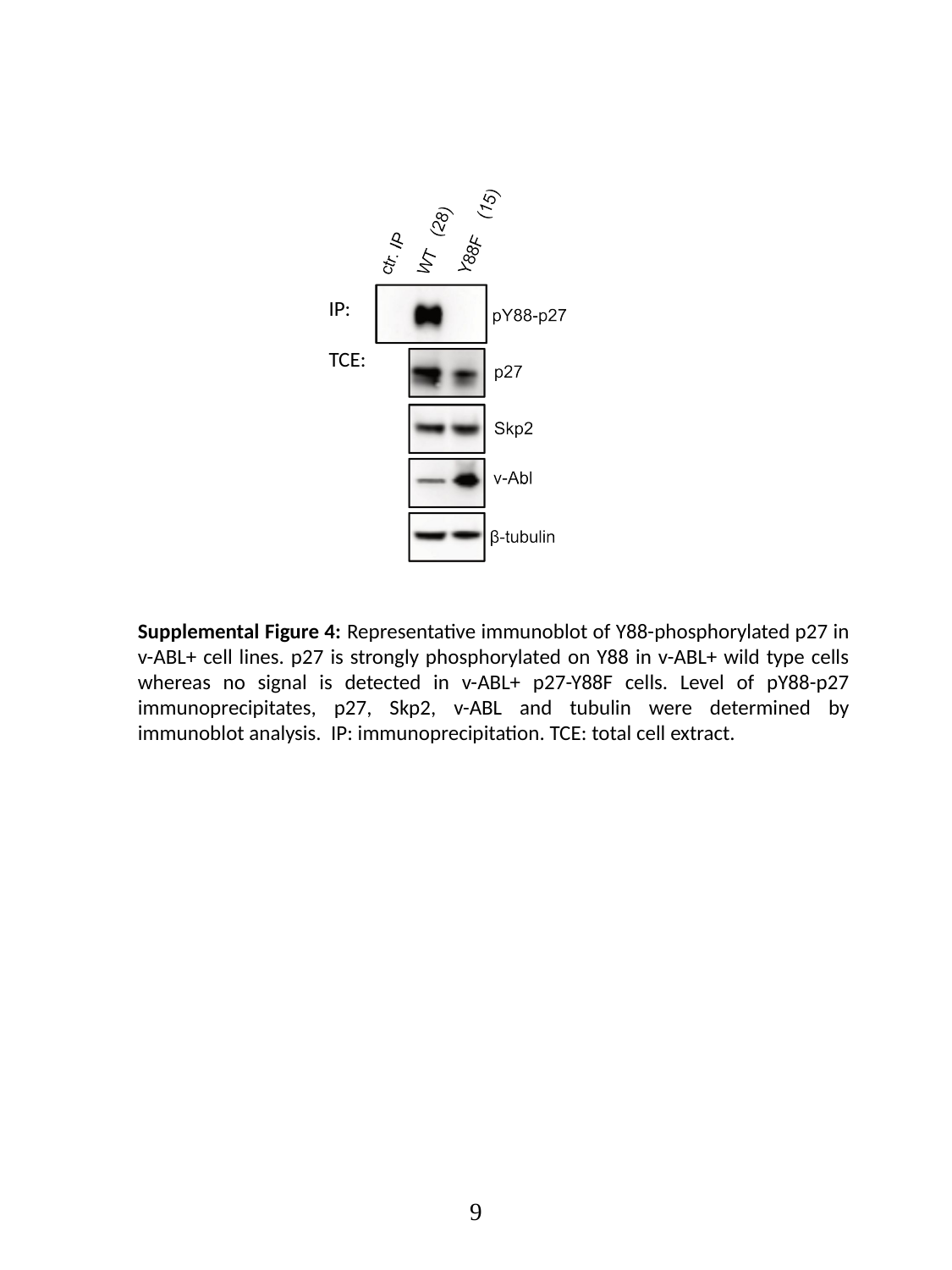

IP:
TCE:
Supplemental Figure 4: Representative immunoblot of Y88-phosphorylated p27 in v-ABL+ cell lines. p27 is strongly phosphorylated on Y88 in v-ABL+ wild type cells whereas no signal is detected in v-ABL+ p27-Y88F cells. Level of pY88-p27 immunoprecipitates, p27, Skp2, v-ABL and tubulin were determined by immunoblot analysis. IP: immunoprecipitation. TCE: total cell extract.
9

## Slide 11
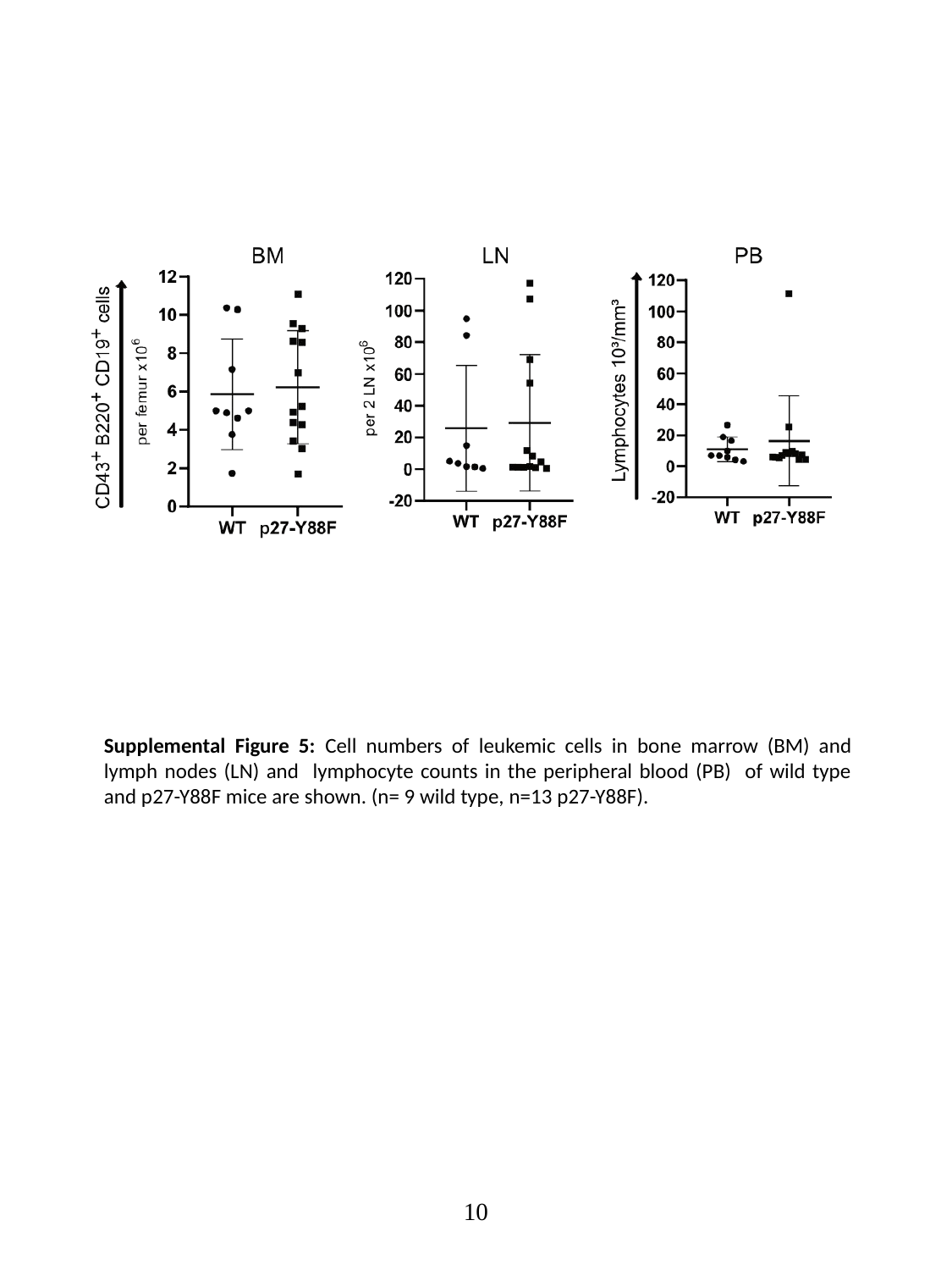

Supplemental Figure 5: Cell numbers of leukemic cells in bone marrow (BM) and lymph nodes (LN) and lymphocyte counts in the peripheral blood (PB) of wild type and p27-Y88F mice are shown. (n= 9 wild type, n=13 p27-Y88F).
10

## Slide 12
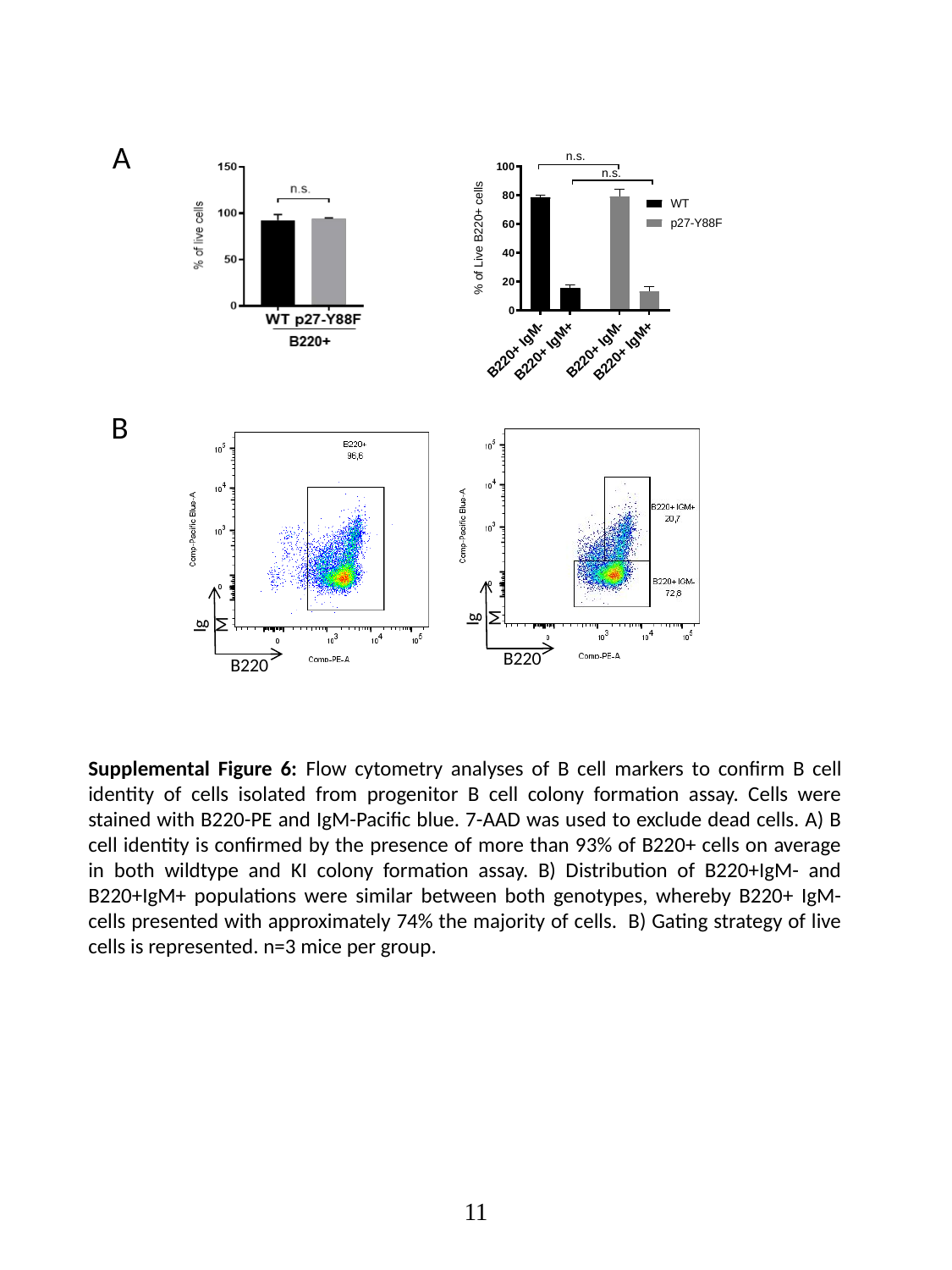

A
B
IgM
B220
IgM
B220
Supplemental Figure 6: Flow cytometry analyses of B cell markers to confirm B cell identity of cells isolated from progenitor B cell colony formation assay. Cells were stained with B220-PE and IgM-Pacific blue. 7-AAD was used to exclude dead cells. A) B cell identity is confirmed by the presence of more than 93% of B220+ cells on average in both wildtype and KI colony formation assay. B) Distribution of B220+IgM- and B220+IgM+ populations were similar between both genotypes, whereby B220+ IgM- cells presented with approximately 74% the majority of cells. B) Gating strategy of live cells is represented. n=3 mice per group.
11

## Slide 13
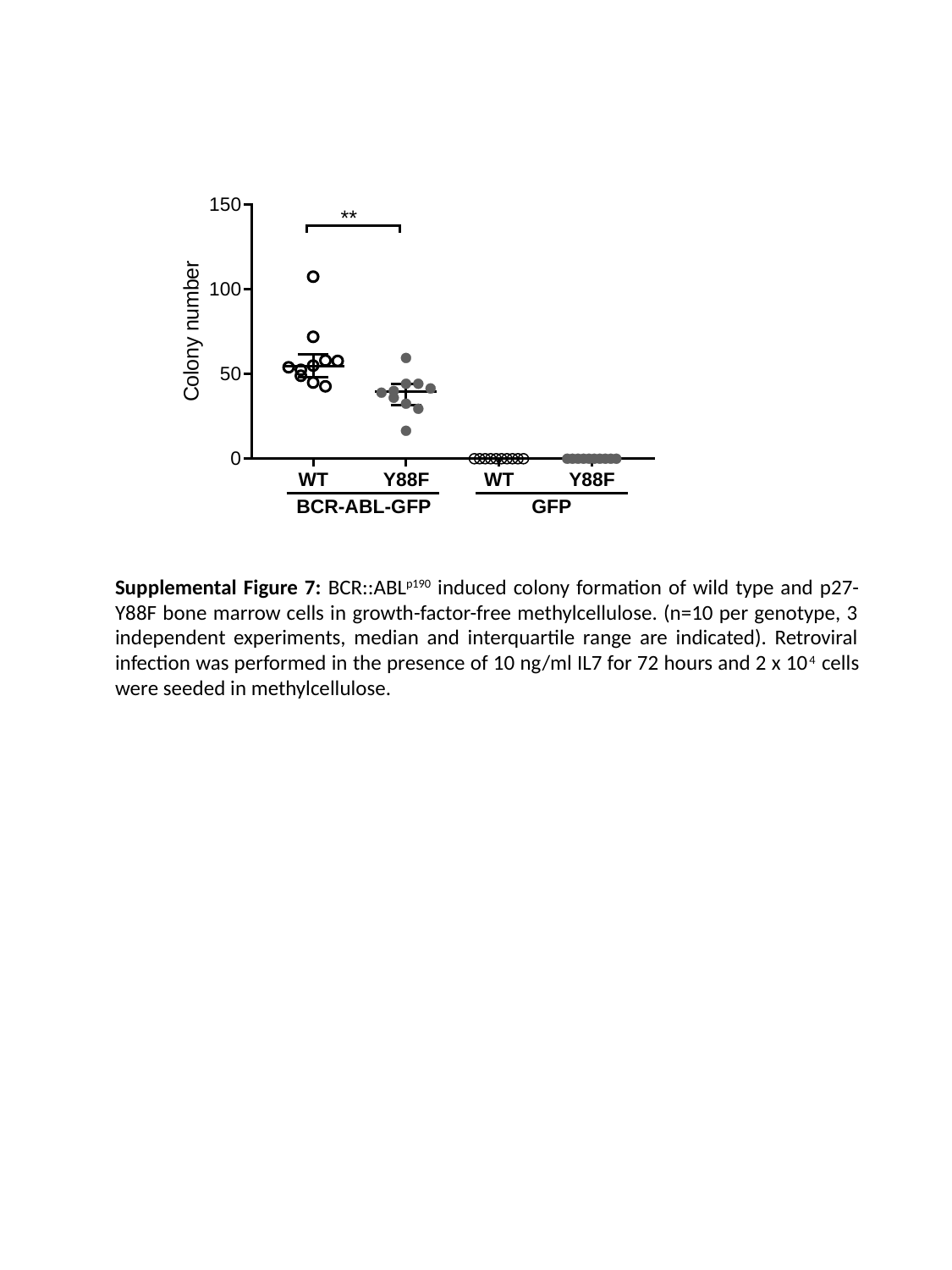

BCR-ABL-GFP
GFP
Supplemental Figure 7: BCR::ABLp190 induced colony formation of wild type and p27-Y88F bone marrow cells in growth-factor-free methylcellulose. (n=10 per genotype, 3 independent experiments, median and interquartile range are indicated). Retroviral infection was performed in the presence of 10 ng/ml IL7 for 72 hours and 2 x 10^4^ cells were seeded in methylcellulose.

## Slide 14
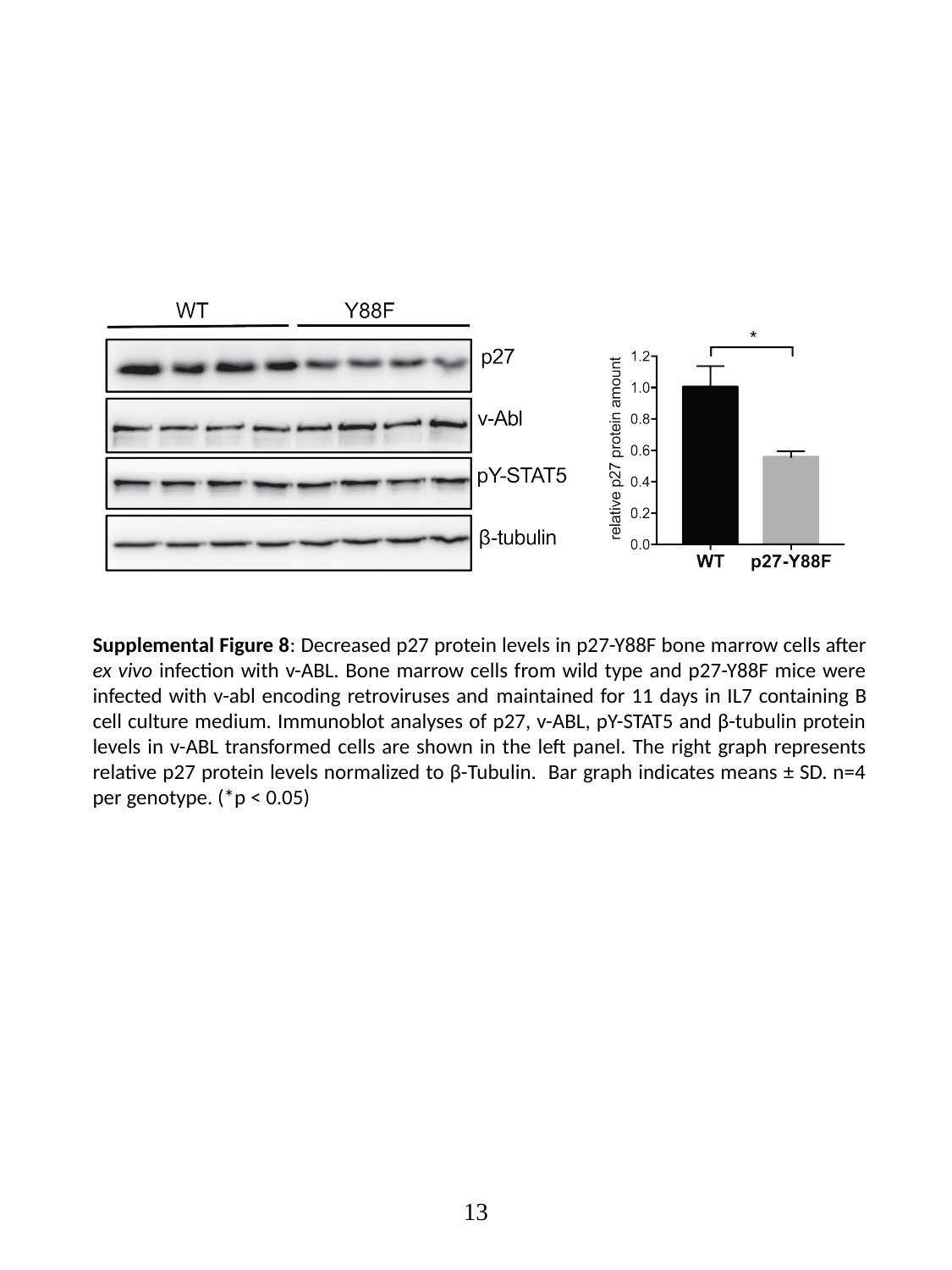

Supplemental Figure 8: Decreased p27 protein levels in p27-Y88F bone marrow cells after ex vivo infection with v-ABL. Bone marrow cells from wild type and p27-Y88F mice were infected with v-abl encoding retroviruses and maintained for 11 days in IL7 containing B cell culture medium. Immunoblot analyses of p27, v-ABL, pY-STAT5 and β-tubulin protein levels in v-ABL transformed cells are shown in the left panel. The right graph represents relative p27 protein levels normalized to β-Tubulin. Bar graph indicates means ± SD. n=4 per genotype. (*p < 0.05)
13

## Slide 15
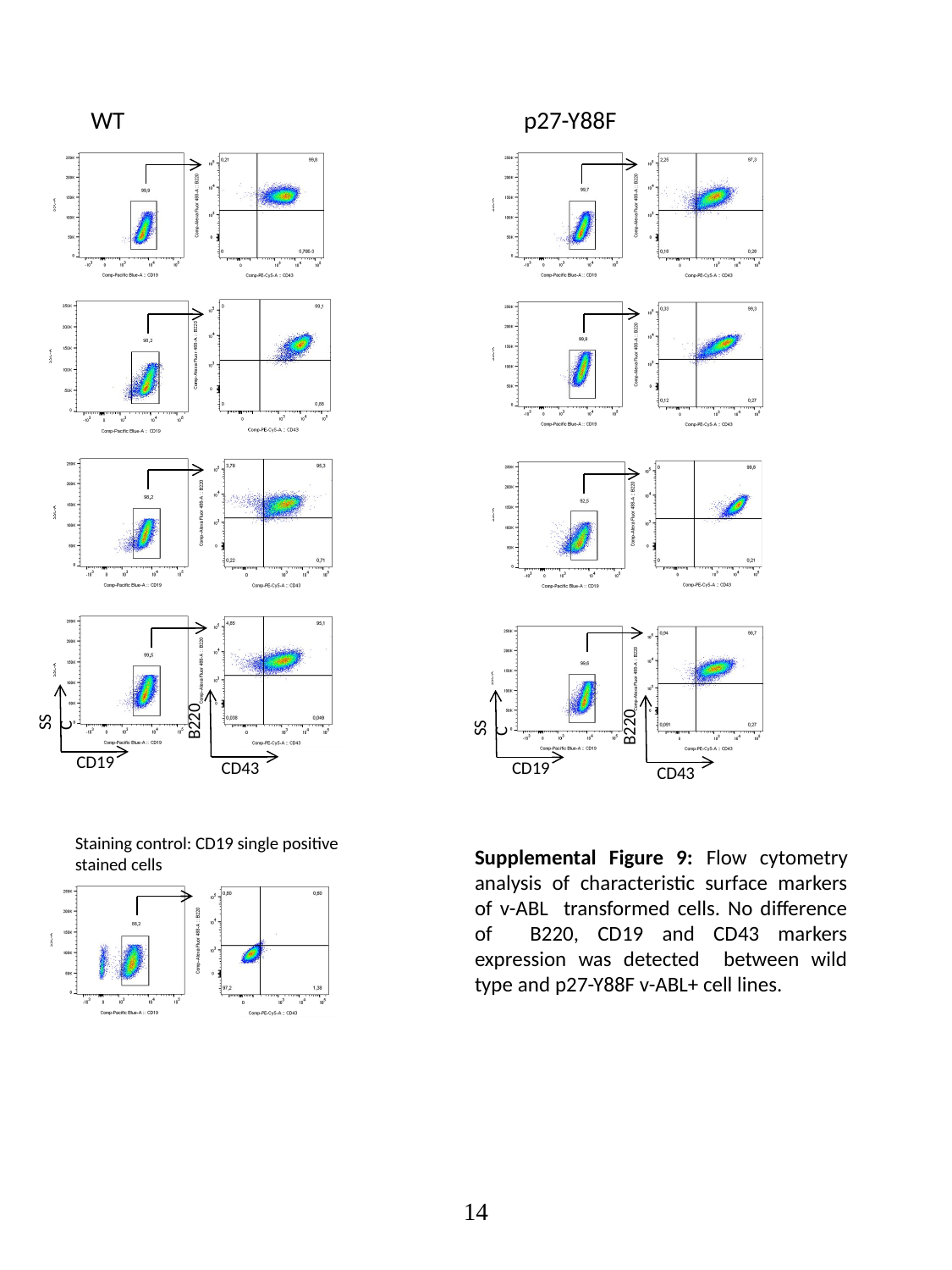

p27-Y88F
SSC
CD19
B220
CD43
WT
SSC
CD19
B220
CD43
Staining control: CD19 single positive stained cells
Supplemental Figure 9: Flow cytometry analysis of characteristic surface markers of v-ABL transformed cells. No difference of B220, CD19 and CD43 markers expression was detected between wild type and p27-Y88F v-ABL+ cell lines.
14

## Slide 16
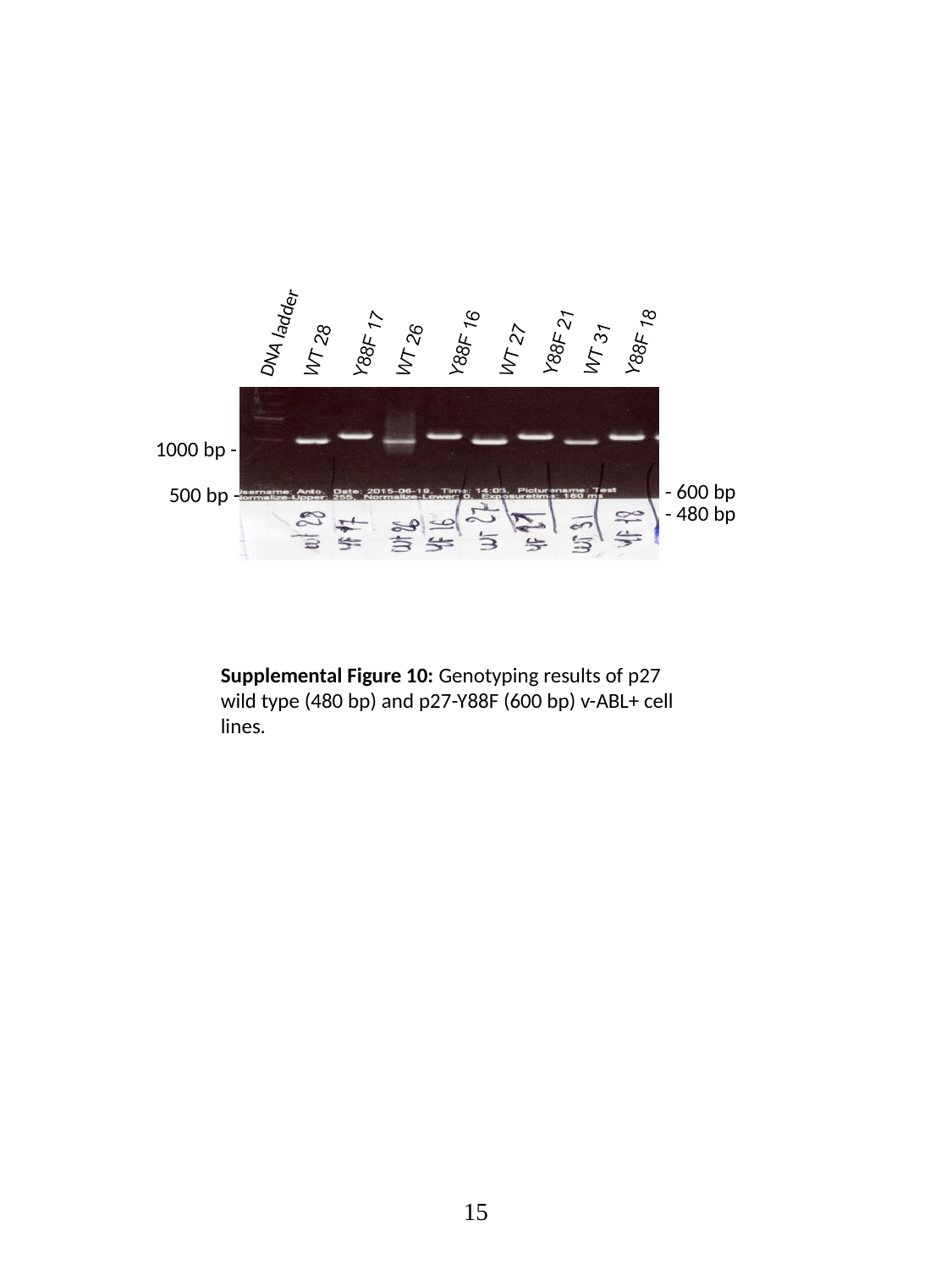

DNA ladder
1000 bp -
- 600 bp
500 bp -
- 480 bp
Supplemental Figure 10: Genotyping results of p27 wild type (480 bp) and p27-Y88F (600 bp) v-ABL+ cell lines.
15

## Slide 17
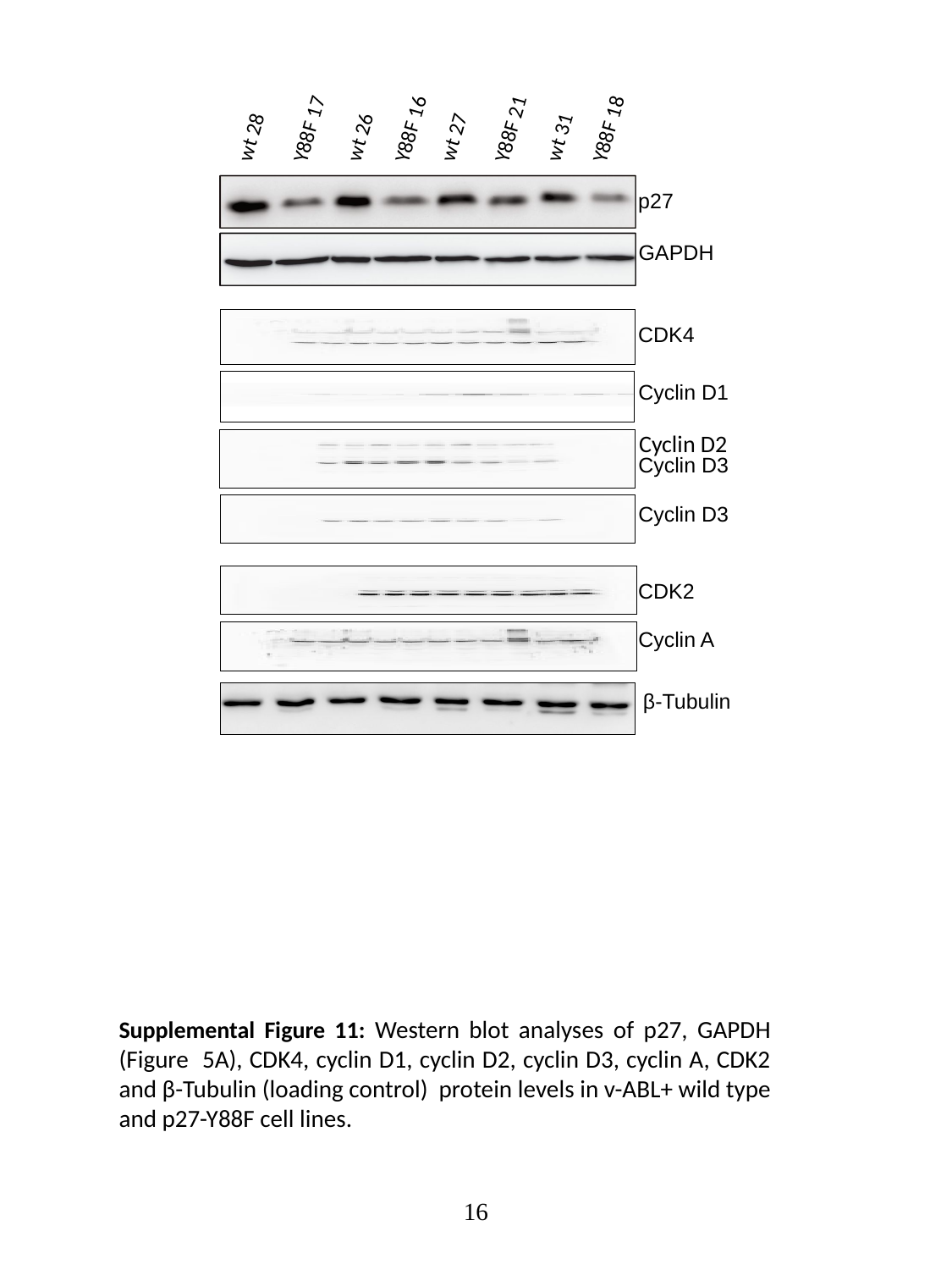

Y88F 17
Y88F 16
Y88F 21
Y88F 18
wt 26
wt 28
wt 27
wt 31
p27
GAPDH
CDK4
Cyclin D1
Cyclin D2
Cyclin D3
Cyclin D3
CDK2
Cyclin A
β-Tubulin
Supplemental Figure 11: Western blot analyses of p27, GAPDH (Figure 5A), CDK4, cyclin D1, cyclin D2, cyclin D3, cyclin A, CDK2 and β-Tubulin (loading control) protein levels in v-ABL+ wild type and p27-Y88F cell lines.
16

## Slide 18
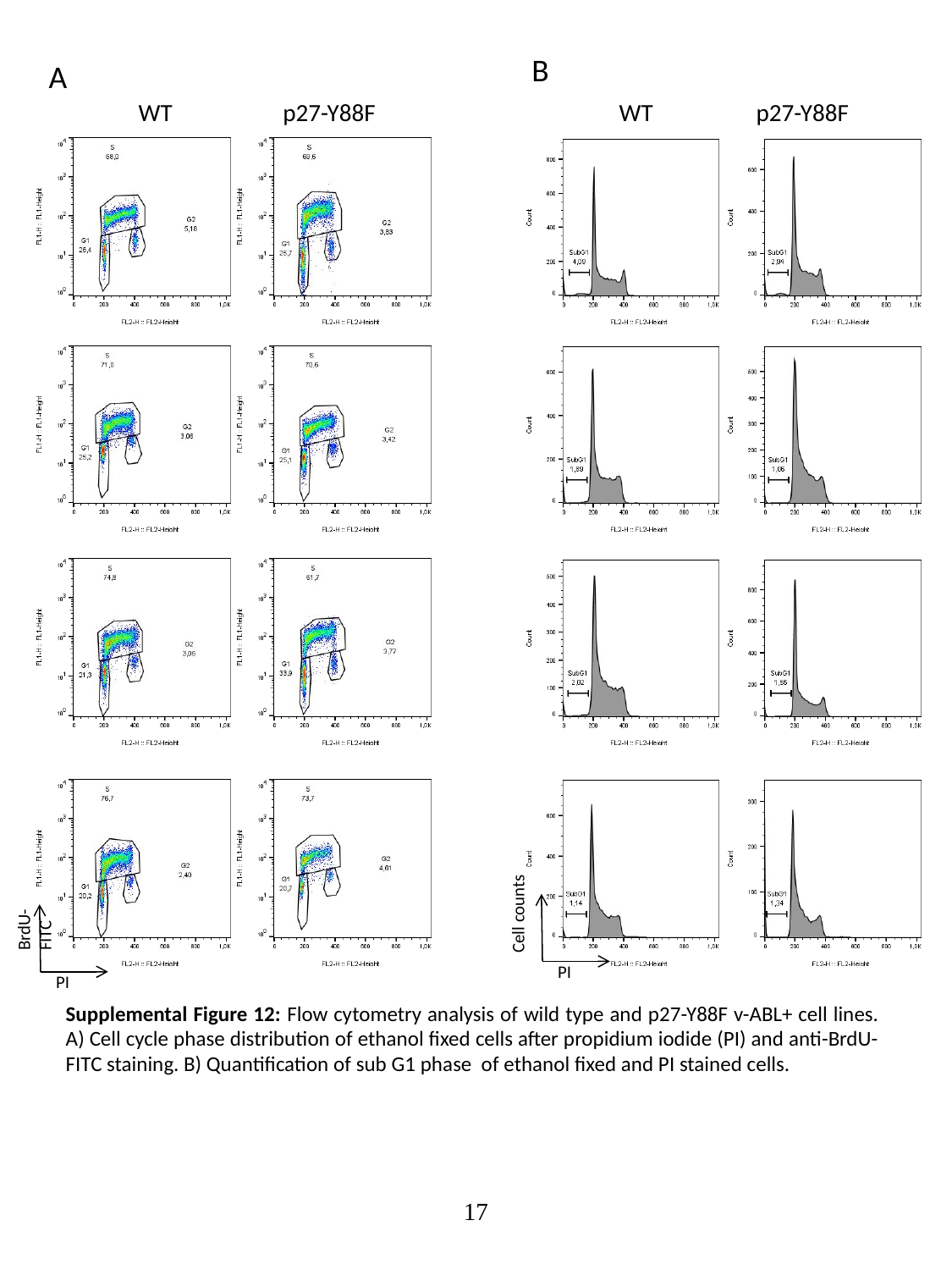

B
A
WT
p27-Y88F
WT
p27-Y88F
Cell counts
PI
BrdU-FITC
PI
Supplemental Figure 12: Flow cytometry analysis of wild type and p27-Y88F v-ABL+ cell lines. A) Cell cycle phase distribution of ethanol fixed cells after propidium iodide (PI) and anti-BrdU-FITC staining. B) Quantification of sub G1 phase of ethanol fixed and PI stained cells.
17

## Slide 19
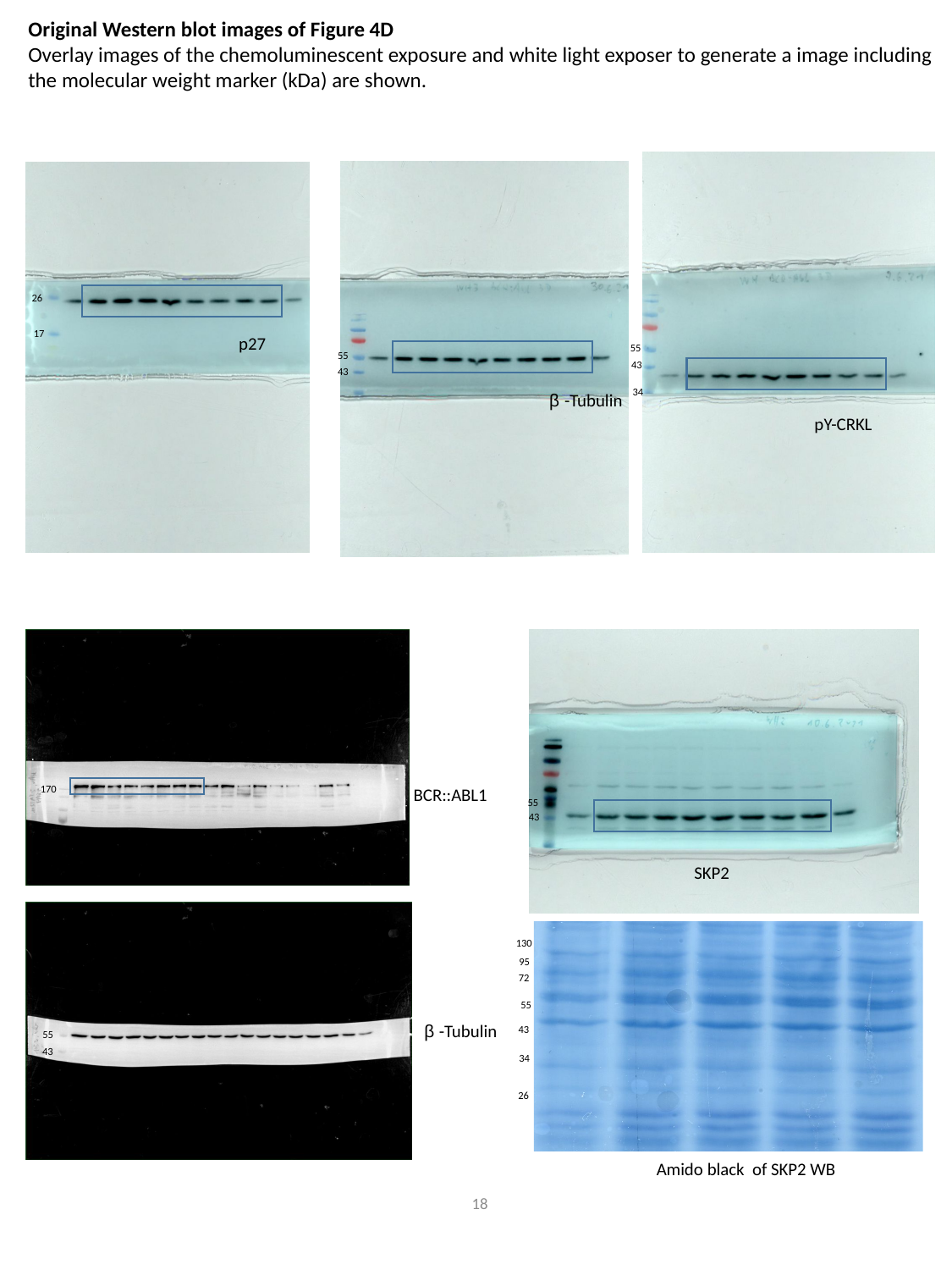

Original Western blot images of Figure 4D
Overlay images of the chemoluminescent exposure and white light exposer to generate a image including the molecular weight marker (kDa) are shown.
26
17
p27
55
55
43
43
34
β -Tubulin
pY-CRKL
170
BCR::ABL1
55
43
SKP2
130
95
72
55
β -Tubulin
43
55
43
34
26
Amido black of SKP2 WB
18

## Slide 20
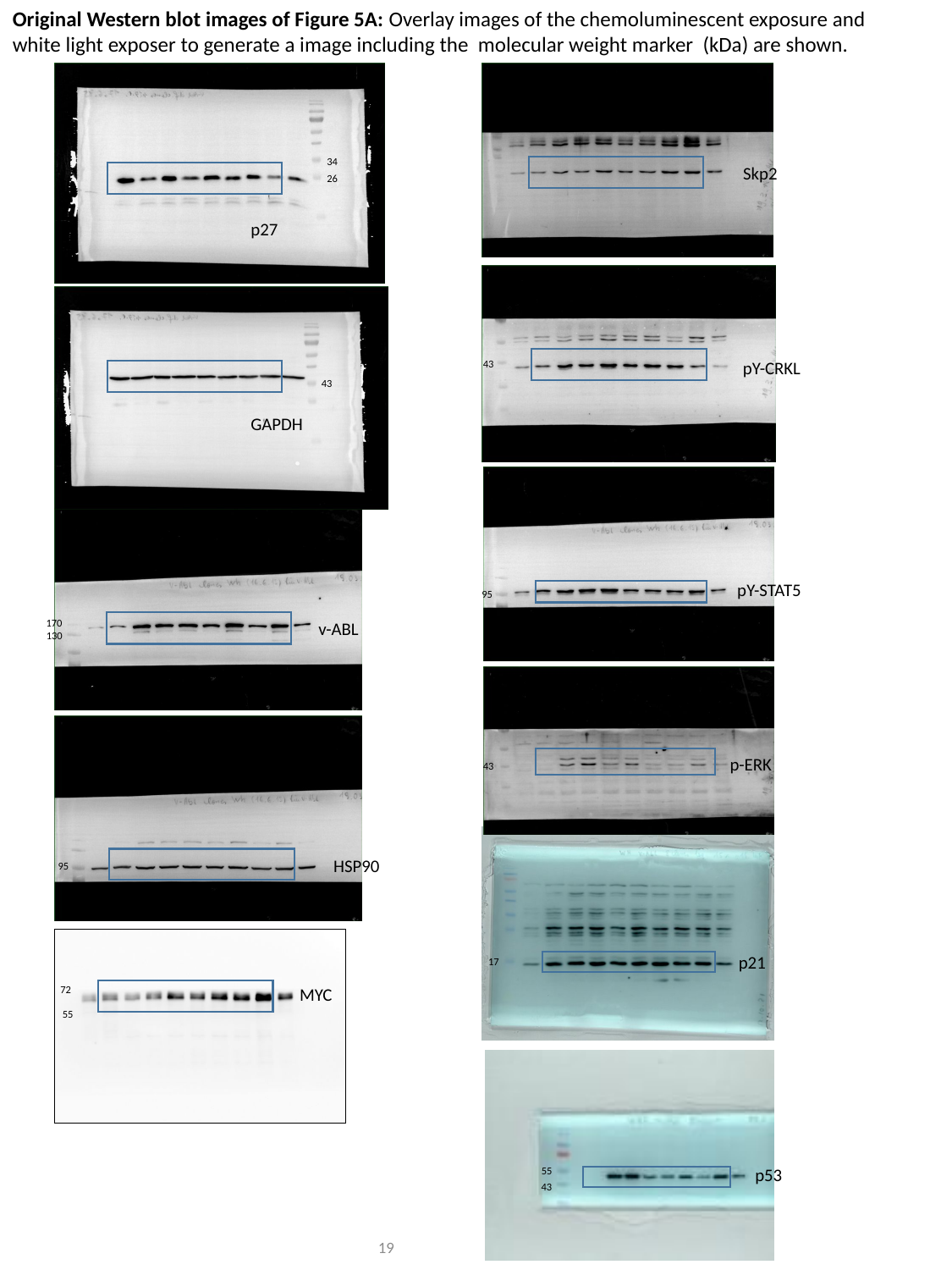

Original Western blot images of Figure 5A: Overlay images of the chemoluminescent exposure and white light exposer to generate a image including the molecular weight marker (kDa) are shown.
34
Skp2
26
p27
pY-CRKL
43
43
GAPDH
pY-STAT5
95
170
v-ABL
130
p-ERK
43
HSP90
95
p21
17
72
MYC
55
p53
55
43
19

## Slide 21
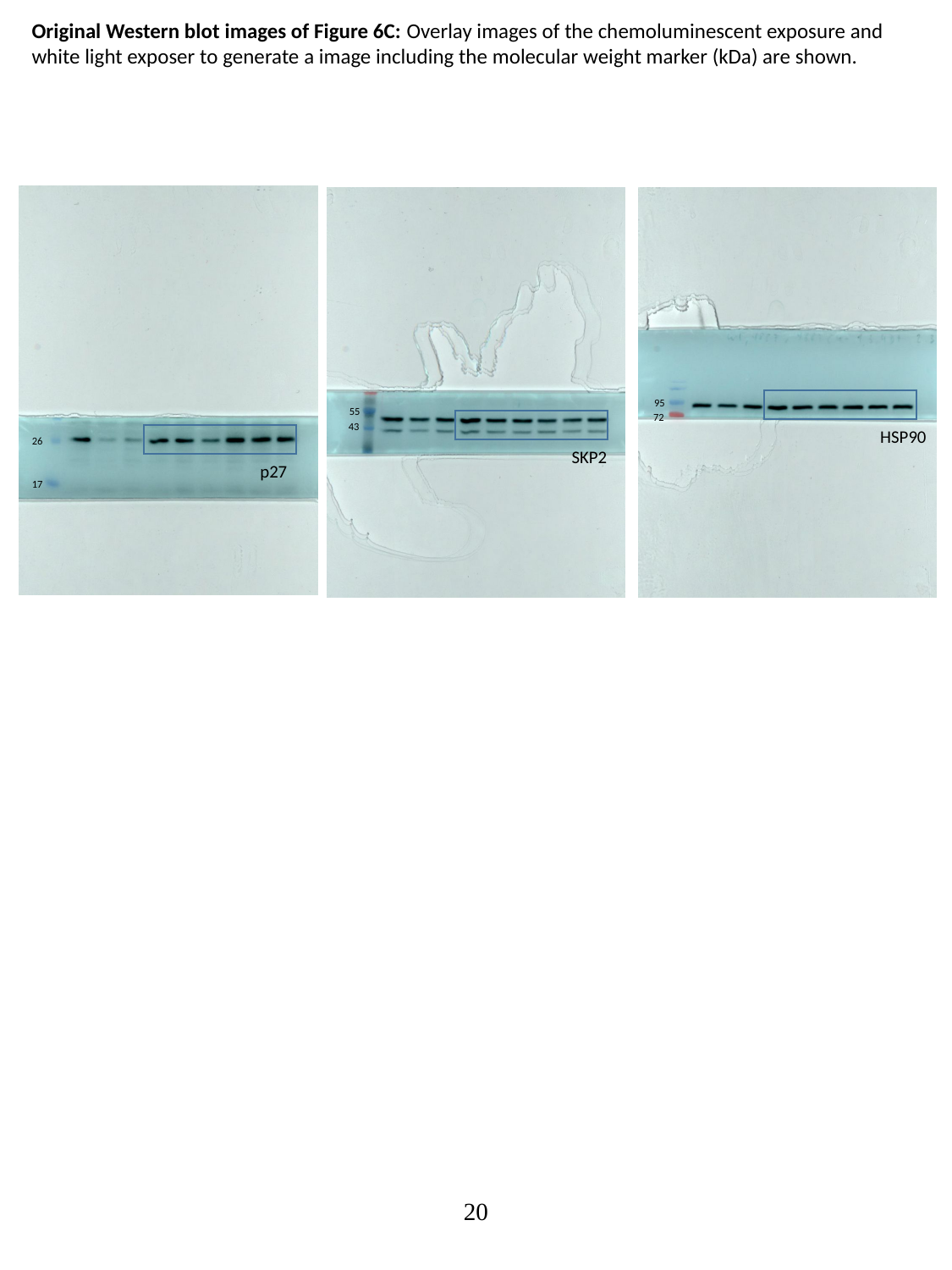

Original Western blot images of Figure 6C: Overlay images of the chemoluminescent exposure and white light exposer to generate a image including the molecular weight marker (kDa) are shown.
95
55
72
43
HSP90
26
SKP2
p27
17
20
